# Nrf2 Is a Central Regulator of Metabolic Reprogramming of Myeloid-Derived Suppressor Cells in Steady State and Sepsis

**DOI:** 10.3389/fimmu.2018.01552

**Published:** 2018-07-06

**Authors:** Kim Ohl, Athanassios Fragoulis, Patricia Klemm, Julian Baumeister, Wiebke Klock, Eva Verjans, Svenja Böll, Julia Möllmann, Michael Lehrke, Ivan Costa, Bernd Denecke, Angela Schippers, Johannes Roth, Norbert Wagner, Christoph Wruck, Klaus Tenbrock

**Affiliations:** ^1^Department of Pediatrics, Medical Faculty, RWTH Aachen, Aachen, Germany; ^2^Department of Anatomy and Cell Biology, Medical Faculty, RWTH Aachen, Aachen, Germany; ^3^Department of General Visceral and Transplantation Surgery, Molecular Tumor Biology, Medical Faculty, RWTH Aachen, Aachen, Germany; ^4^Institute of Pharmacology and Toxicology, RWTH Aachen, Aachen, Germany; ^5^Department of Medicine I, Medical Faculty, RWTH Aachen, Aachen, Germany; ^6^Interdisciplinary Centre for Clinical Research (IZKF) Aachen, Medical Faculty, RWTH Aachen, Aachen, Germany; ^7^Institute of Immunology, University of Münster, Münster, Germany

**Keywords:** Nrf2, myeloid-derived suppressor cell, LPS, sepsis, ROS

## Abstract

Arising in inflammatory conditions, myeloid-derived suppressor cells (MDSCs) are constantly confronted with intracellular and extracellular reactive oxygen species molecules and oxidative stress. Generating mice with a constitutive activation of Nuclear factor (erythroid-derived 2)-like 2 (Nrf2) we show a pivotal role of the antioxidant stress defense for development of these immune-modulatory cells. These mice are characterized by a massive increase of splenic CD11b^+^Gr-1^+^ cells, which exhibit typical suppressive characteristics of MDSCs. Whole transcriptome analysis revealed Nrf2-dependent activation of cell cycle and metabolic pathways, which resemble pathways in CD11b^+^Gr-1^+^ MDSCs expanded by *in vivo* LPS exposure. Constitutive Nrf2 activation thereby regulates activation and balance between glycolysis and mitochondrial metabolism and hence expansion of highly suppressive MDSCs, which mediate protection in LPS-induced sepsis. Our study establishes Nrf2 as key regulator of MDSCs and acquired tolerance against LPS-induced sepsis.

## Introduction

Myeloid-derived suppressor cells (MDSCs) are a heterogeneous population of immature myeloid cells (IMCs), induced under pathological conditions such as infection and sepsis, chronic inflammation, and cancer (1). The main feature of these cells is their potent immunosuppressive activity. It is therefore not surprising that MDSCs have emerged as major regulators of pathogenic and inflammatory immune responses (2). In addition to its important role in cancer, MDSCs expand during murine sepsis as well as in septic patients (3, 4). Although sepsis patients have high levels of inflammatory mediators, components of their immune system are suppressed as well. This modified steady state of innate immunity after infection is referred to as innate memory (5, 6). Innate memory is based on epigenetic reprogramming which is broadly defined as sustained change in transcription programs and cell physiology (5, 7). Induction of innate memory is thus accompanied by significant changes in cellular metabolism. When inappropriately activated, innate memory programs can become maladaptive as in post-sepsis immune paralysis, which is associated with severe energy metabolism defects of leukocytes (8). Molecular mechanism that mediate innate memory at the level of cell types and the immunological metabolic and epigenetic processes behind are therefore an important area of research. By suppressing innate as well as adaptive immune responses, MDSCs have protective roles in the initial hyper-inflammatory reaction, however, are also involved in sepsis-induced innate immunoparalysis (3, 4, 9–11). We would therefore specify MDSCs as central player in innate memory. However, there still remain open questions about, how expansion and functions of MDSCs are regulated in sepsis and how metabolic dysregulations of MDSCs affect innate immunity in sepsis.

Cellular metabolism and oxidative stress are intimately linked, immune cells are constantly confronted with intracellular and extracellular reactive oxygen species (ROS) molecules in steady state and moreover in inflammatory conditions. During sepsis, enhanced levels of ROS molecules, either produced by NADPH oxidases during oxidative bursts or by mitochondrial dysfunction, lead to oxidative stress conditions (12). Interestingly immune cell types vary with regard to their ROS susceptibility and although ROS are toxic to most cells, MDSC survive despite their elevated content and release of ROS (13). Moreover, high numbers of MDSCs arise in oxidative stress prone conditions such as inflammation, infection, and cancer.

This prompted us to analyze ROS-mediated signaling pathways in myeloid cells in steady state and sepsis. We hereby identified Nuclear factor (erythroid-derived 2)-like 2 (Nrf2), the transcriptional regulator of the antioxidant stress defense, as key regulator of metabolic reprogramming of MDSCs.

## Materials and Methods

### Mice Strains

Experiments were performed with age-matched WT, Keap^fl/fl^, *VAV^cre^Keap^fl/fl^*, and *Nrf2*^−/−^ mice (all C57BL/6). *VAV^cre^Keap^fl/fl^* mice were generated by crossing Keap1-flox mice (14) with VAVcre mice. *VAV^cre^*^−^*Keap^fl/fl^* mice were used as controls (denoted as *Keap^fl/fl^*). *Nrf2*^−/−^ mice have been described previously (15) and were bred in our animal facility and kept under standardized conditions, as were the OT-II mice, CD45.1 congenic mice (C57BL/6), and RAG^−/−^ mice used.

### LPS Treatment

8- to 10-week-old mice with at least 25 g were used for this study. Treatments were conducted with either vehicle + lethal dose 5 mg/kg BW LPS (sepsis group) or low dose + lethal doses 30 mg/kg BW LPS (tolerance group) in 250 µl 0.9% NaCl i.p. All animals were monitored thrice per day in 6 h intervals to document weight and body temperature.

Rapamycin was first solved in ethanol and then diluted in 5.2% PEG/Tween in NaCl. Mice received either vehicle (5.2% PEG/Tween) or 2 mg/kg of body weight rapamycin by daily i.p. injections.


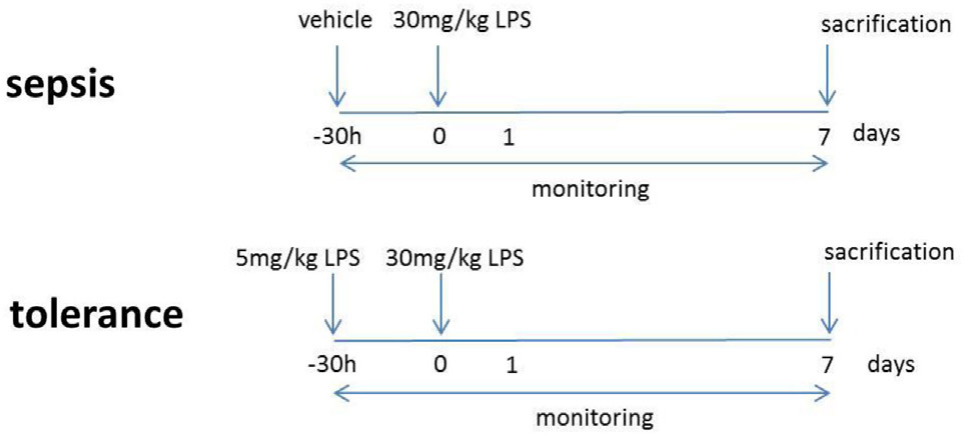


### Transfer Colitis

To induce transfer colitis, RAG2^−/−^ mice were adoptively transferred with either 2 × 10^6^ CD4^+^CD25^−^ T cells alone, with 2 × 10^6^ CD11b^+^Gr-1^+^ cells alone or with both 2 × 10^6^ CD4^+^ T cells and 2 × 10^6^ CD11b^+^Gr1^+^ cells. After 6 weeks, the mice were sacrificed. Spleens and mesenterial lymph nodes (mLNs) were harvested for further analysis. One part of the colon was fixed in formalin for histological scoring and the other part was fixed in RNAlater (Qiagen, Germany) for subsequent mRNA analysis.

### Histological Scoring

4 µm paraffin sections from the fixed colon were cut serially, mounted onto glass slides, and deparaffinized. The colon sections were stained with hematoxylin and eosin by the Core Facility (IZKF) of the RWTH Aachen University. Blinded histological scoring was performed using a standard microscope, based on The Jackson Laboratory Score method as described previously (16, 17). Each colon section was scored for the four general criteria: severity, degree of hyperplasia, degree of ulceration, if present, and percentage of area involved. A subjective range of 1–3 (1 = mild, 2 = moderate, 3 = severe) was used for the first three categories. Severity: focally small or widely separated multifocal areas of inflammation limited to the lamina propria were graded as mild lesions (1). Multifocal or locally extensive areas of inflammation extending to the submucosa were graded as moderate lesions (2). If the inflammation extended to all layers of the intestinal wall or the entire intestinal epithelium was destroyed, lesions were graded as severe (3). Hyperplasia: mild hyperplasia consisted of morphologically normal lining epithelium that was at least twice as thick (length of crypts) as adjacent or control mucosa. Moderate hyperplasia was characterized by the lining epithelium being two or three times normal thickness, cells were hyperchromatic, numbers of goblet cells were decreased, and scattered individual crypts developed an arborizing pattern. Severe hyperplastic regions exhibited markedly thickened epithelium (four or more times normal thickness), marked hyperchromasia of cells, few to no goblet cells, a high mitotic index of cells within the crypts, and numerous crypts with arborizing pattern. Ulceration was graded as: 0 = no ulcer, 1 = 1–2 ulcers (involving up to a total of 20 crypts), 2 = 1–4 ulcers (involving a total of 20–40 crypts), and 3 = any ulcers exceeding the former in size. A 10% scale was used to estimate the area involved in the inflammatory process (0 = 0%, 1 = 10–30%, 2 = 40–70%, 3 = >70%).

### BrdU Assay

Mice were fed orally with 0.8 mg/ml BrdU (BD) in drinking water. Drinking water was changed every 2 days. After 14 days mice were sacrificed, spleens and BMDCs were harvested, and BrdU incorporation was assessed by flow cytometry according to the manufacturer’s instructions (BrdU Flow, Kit, BD).

### Mixed Bone Marrow Chimeras

BM cells were isolated from femurs and tibias of age-matched donor animals (WT CD45.1 and *VAV^cre^Keap^fl/fl^* CD45.2). RAG2^−/−^ mice were lethally irradiated (2 Gy × 6.8 Gy) and co-injected with 5 × 10^6^ cells of each genotype after irradiation, or injected with 10 × 10^6^ cells of only one genotype (WT CD45.1 or *VAV^cre^Keap^fl/fl^* CD45.2 cells). The mice received antibiotic treatment for 14 days [40 µl Borgal-solution (24%)/100 ml drinking water]. Eight weeks later, the mice were sacrificed and spleens analyzed by flow cytometry.

### Cell Isolation

Mouse BM cells were flushed from femurs and tibias with Dulbecco medium. Erythrocytes were lysed with lysis buffer (eBioscience) for 3 min at room temperature, and the remaining cells were washed once with PBS. Single cell suspensions were isolated from spleens and erythrocytes were lysed with lysis buffer. MDSCs were isolated from splenocytes by magnetic cell separation (Miltenyi, Germany). Flow cytometric analysis revealed high purity (90%) of isolated CD11b^+^Gr-1^+^ cells. CD4^+^ cells were isolated by magnetic cell separation using the CD4^+^ T cell isolation kit (Miltenyi), while CD4^+^CD25^+^ Treg cell isolation kits (Miltenyi) were used to isolate CD4^+^CD25^−^ cells and perform adoptive transfer colitis.

### Flow Cytometry

For surface staining, single cell suspensions were stained with anti-CD11b, anti-Gr-1, anti-CD4, anti-CD3, anti-CD8, anti-CD25, anti-CD19, anti-CD11c, anti-F4/80, anti-CD45.1, and anti-CD45.2 (all from eBioscience, Germany). To analyze Foxp3, pS6, p4EBP-1, Nos2, p-mTOR, and arginase expression, cells were fixed and permeabilized with a FOXP3 staining buffer set (eBioscience, Germany) following the manufacturer’s instructions and stained with anti-Foxp3 antibodies (eBioscience, Germany), anti pS6, p4EBP-1 (BD Biosciences), anti-p-mTOR (ebioscience, Germany), anti-arginase and sheep-IgG (both R&D), or anti-NOS2 and mouse-IgG2a (both eBiosience) antibodies for 30 min. To analyze mitochondrial mass by flow cytometry, cells were incubated with 25 ng/ml nonyl acridine orange (Thermo Fischer Scientific) for 10 min at 37°C and maintained on ice until flow cytometric analysis. Glucose uptake was determined by means of a glucose uptake cell-based kit (Cayman Chemical). 2 × 10^6^ cells/ml were incubated in glucose-free medium for 2 h. Afterwards 100 µg/ml 2-NBDG was added and incubation continued in a cell incubator at 37°C. Incubation was stopped by immediate transfer of cell culture plates to 4°C conditions. Cells were washed with a cell-based assay buffer according to the manufacturer’s instructions and kept at 4°C until flow cytometric analysis. A total reactive oxygen species assay kit (eBioscience) was used to identify ROS, following the manufacturer’s instructions. In detail, this involved incubation of the cells with ROS assay stain for 60 min at 37°C, washing once with PBS and analysis on the flow cytometer. To identify apoptotic cells, cells were first labeled with cell viability dye (eBioscience) and then incubated with fluorochrome conjugated Annexin-V (eBioscience) in Annexin-V binding buffer according to the manufacturer’s instructions. BrdU staining was performed according to the manufacturer’s protocol with BrdU Flow Kit (BD Pharmingen). 7-AAD staining was performed by adding 7-AAD (BD Pharmingen) directly to the cells before measurement.

Flow cytometry was carried out using FACSCanto II device (BD Biosciences, Germany). Data analysis was performed using FCS Express Software.

### RNA Isolation and Real-Time PCR

Total RNA from isolated MDSCs and colon tissue was isolated using the RNeasy Mini Kit (Qiagen, Germany). cDNA was then generated from 200 ng total RNA using the RevertAid H Minus First Strand cDNA Synthesis Kit (Thermo Fisher Scientific, USA) according to the manufacturer’s instructions. RT-PCR was performed using the SYBR Green PCR kit (Eurogentec, Germany) and data were acquired with the ABI prism 7300 RT-PCR system (Applied Biosystems/Life Technologies, Germany). Each measurement was set up in duplicate. After normalization to the endogenous reference control gene β-actin for mice, the relative expression was calculated. The sequences of primers used in this study are listed in Table S1 in Supplementary Material.

### Seahorse Assay

2 × 10^5^ cells were seeded on gelatin-coated plates and OCR/ECAR measured using the XF96 Extracellular Flux Analyzer (Seahorse Bioscience) following the manufacturer’s instructions. OCR was measured in XF media containing 11 mmol/l glucose and 1 mmol/l sodium pyruvate under basal conditions and in response to 1 µmol/l oligomycin, 1 µmol/l carbonyl cyanide p-trifluoromethoxyphenylhydrazone (FCCP), and 0.1 µmol/l rotenone plus 0.1 µmol/l antimycin A. Extracellular acidification rate (ECAR) was measured in assay medium (XF Media supplemented with 4.5 g/l glucose and 2 mM glutamine) under basal conditions and in response to 10 mM glucose, 1 M oligomycin, and 100 mM 2-deoxyglucose.

### *In Vitro* MDSC Generation

2 × 10^6^ murine bone marrow cells per ml were cultured in RPMI with 2 g/l glucose supplemented with 10% heat-inactivated FCS (Life Technologies). In some experiments, glucose concentrations were adapted as indicated. To obtain BM-derived MDSCs, medium was supplemented with IL-6 (10 ng/ml) and GM-CSF (20 ng/ml) (both Peprotech). On day 3 of culture, the original medium was replaced with fresh medium containing cytokines and cultures were maintained at 37°C in 5% CO_2_-humidified atmosphere for an additional 3 days. To analyze effects of rapamycin, 1 µM rapamycin (Cayman Chemical) were added at day 0 and 3.

To analyze dimethyl fumarate (DMF) effects on human cells, 2 × 10^6^ human PBMCs per ml were cultured in RPMI in the presence or absence of 2 µg/ml DMF.

### Suppression Assays

DCs were generated by culturing BM cells in the presence of GM-CSF (50 ng/ml) and IL-4 (40 ng/ml) for 6 days. Cells were fed with OVA peptide (1 µM) for 2 h and extensively washed with PBS. CD4^+^ OT-II cells were isolated by magnetic cell separation and labeled with cell proliferation dye (5 µM) (eBioscience) according to the manufacturer’s instructions. DCs and CD4^+^ T cells were co-cultured in a 1:10 ratio in U-bottom 96-well plates. MDSCs were isolated by magnetic cell isolation and were added to DC/T cell cultures in ratios of 1:1 or 1:0.5. After 3 days, proliferation of CD4 T cells was assessed by flow cytometry.

### RNA Extraction and Microarray for Gene Expression Analysis

Genome wide transcriptome analyses for *VAV^cre^Keap^fl/fl^* and WT (*VAV^cre^*^−^*Keap^fl/fl^*) MDSCs were performed in independent triplicates using Gene Chip^®^ Mouse Gene 2.0 arrays (Affymetrix, Santa Clara, CA, USA). Total RNA extraction was carried out using the RNeasy Micro Kit (Qiagen, Germany) according to the manufacturer’s protocol and then quantified (Nanodrop). RNA quality was assessed using the RNA 6000 Nano Assay with the 2100 Bioanalyzer (Agilent, Santa Clara, CA, USA). Samples for the Gene 2.0 arrays were prepared and hybridized to the arrays according to the Affymetrix WT Plus Kit manual. Briefly, for each sample, 100 ng of total RNA was reversed transcribed into cDNA using a random hexamer oligonucleotide tagged with a T7 promoter sequence. After second strand synthesis, double strand cDNA was used as a template for amplification with T7 RNA polymerase to obtain antisense cRNA. Random hexamers and dNTPs spiked out with dUTP were then used to reverse transcribe the cRNA into single stranded sense strand cDNA. The cDNA was then fragmented with uracil DNA glycosylase and apurinic/apyrimidic endonuclease 1. Fragment size was checked using the 2100 Bioanalyzer and ranged from 50 to 200 bp. Fragmented sense cDNA was biotin-endlabeled with TdT and probes were hybridized to the Gene 2.0 arrays at 45°C for 16 h with 60 rpm. Hybridized arrays were washed and stained on a Fluidics Station 450 (program: FS450 0002) and scanned on a GeneChip^®^ Scanner 3000 7 G (both Affymetrix). Raw image data were analyzed with Affymetrix^®^ Expression Console™ Software (Affymetrix, USA), and gene expression intensities were normalized and summarized with a robust multiarray average algorithm (18). Transcripts that were expressed differently more than 1.5-fold with a raw *p*-value lower than 0.05 between the sample groups were categorized as regulated. Enrichment analysis for Wiki pathways was performed using WebGestalt (19). For the enrichment analysis, only genes changed at least 1.5-fold with a *p*-value lower than 0.05 between *VAV^cre^Keap^fl/fl^* and WT (*VAV^cre^*^−^*Keap^fl/fl^*) samples were taken into consideration.

### Statistical Analysis

All data are presented as mean ± SEM or SD if indicated. Differences between two groups were evaluated using two-tailed, unpaired or paired (if indicated) Student’s *t*-test. All statistical analysis and subsequent graphics generation were performed using GraphPad Prism version 7.0 (GraphPad Software, USA). A *p*-value <0.05 was considered to be statistically significant.

### Study Approval

The study was approved by the regional government authorities and animal procedures were performed according to German legislation for animal protection. Permission for the projects was granted by the Regierungspräsident/LANUV Nordrhein-Westfalen.

## Results

### *VAV^cre^Keap^fl/fl^* Mice Develop Splenomegaly Due to an Accumulation of CD11b^+^Gr-1^+^ Cells

**Figure 1:**
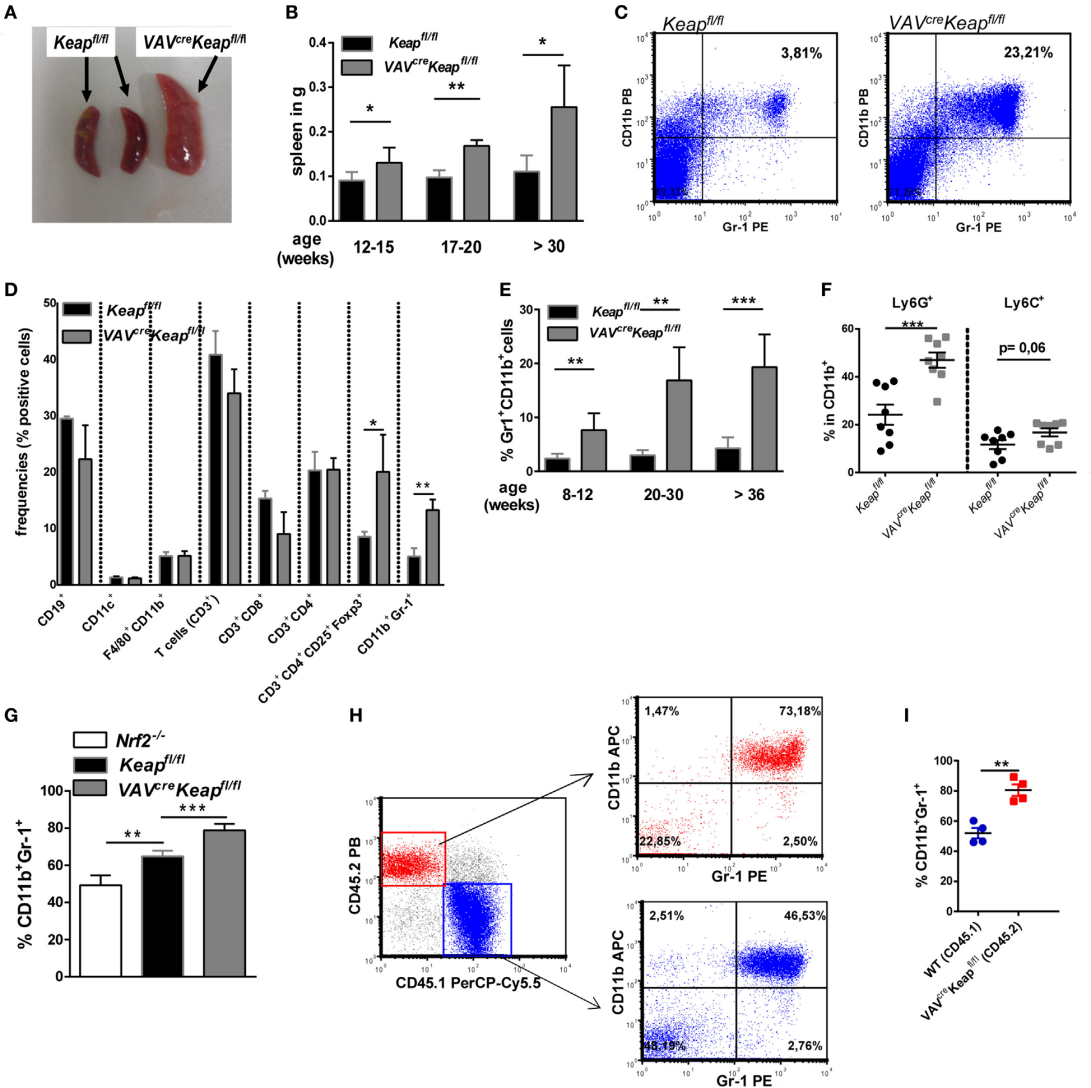
Nrf2 activation enhances CD11b^+^ Gr-1^+^ cells. **(A)** Spleens from old (50–52 weeks) *Keap^fl/fl^* and *VAV^cre^Keap^fl/fl^* mice. **(B)** Weight of spleens from *Keap^fl/fl^* and *VAV^cre^Keap^fl/fl^* mice at different ages. Bars indicate mean ± SD of at least three mice per group. **(C)** Representative dot plot depicting frequencies of CD11b^+^Gr-1^+^ cells in spleens from old (50–52 weeks) *Keap^fl/fl^* and *VAV^cre^Keap^fl/fl^* mice. **(D)** Frequencies of immune cell populations in spleens from 16-week-old *Keap^fl/fl^* and *VAV^cre^Keap^fl/fl^* mice. Bars indicate mean ± SEM of three mice per group. **(E)** Frequencies of CD11b^+^ Gr-1^+^ cells in spleens from *Keap^fl/fl^* and *VAV^cre^Keap^fl/fl^* mice at different ages. Bars indicate mean ± SD of at least six mice per group. **(F)** Frequencies of Ly6G^+^ and Ly6C^+^ cells within splenic CD11b^+^ cells from *Keap^fl/fl^* and *VAV^cre^Keap^fl/fl^* mice. **(G)** BM-derived cells were incubated with GM-CSF and IL-6 and frequencies of CD11b^+^Gr-1^+^ cells were assessed by flow cytometry. Bars indicate mean ± SD of at least three mice per group. **(H)** Mixed BM chimeric mice were analyzed 8 weeks after transfer of *CD45.1 WT* and *CD45.2 VAV^cre^Keap^fl/fl^* BM cells into lethally irradiated RAG2^−/−^ recipient mice. WT mice were used, since *Keap^fl/fl^* CD45.1 mice were not available. Representative dot plots of splenic CD45.2^+^ Gr-1^+^CD11b^+^ and CD45.1^+^ Gr-1^+^CD11b^+^ cells are shown. **(I)** Percentages of splenic CD11b^+^Gr-1^+^ cells of *CD45.1* and *CD45.2* origin in bone marrow chimeras. For **(F,I)**, each symbol indicates an individual mouse. Horizontal lines represent the mean; error bars represent SEM. Two-tailed unpaired *t*-tests were used to determine *p*-values for all statistical analysis.

To analyze oxidative stress signaling in immune cells, we generated a mouse with constitutive Nrf2 activation in all hematopoietic cells by breeding Kelch ECH associating protein 1 (Keap1)-flox mice with VAV-CRE recombinase mice (*Vav^cre^Keap^fl/fl^*). Keap1 suppresses Nrf2 transcriptional activity under basal conditions, thus deletion of Keap1 results in constitutive nuclear accumulation and activation of Nrf2 (20). *Vav^cre^Keap^fl/fl^* mice are born at expected Mendelian ratios, are healthy and cancer-free, and have a normal life span. However, on aging, *Vav^cre^Keap^fl/fl^* mice develop splenomegaly (Figures 1A,B) due to an increase in cell size (Figure S1A in Supplementary Material). Flow cytometric analysis of immune cells in the spleens revealed a specific enrichment of two immunosuppressive cell subsets, namely regulatory T (T_reg_) and CD11b^+^Gr-1^+^ cells (Figures 1C,D). While absolute numbers (Figure S1B in Supplementary Material) and frequencies (Figure 1D) of B cells (CD19^+^), DCs (CD11c^+^), macrophages (CD11b^+^F4/80^+^) and T cells (CD3^+^), as well as cytotoxic T cells (CD3^+^CD8^+^) and T helper cells (CD3^+^CD4^+^) were not raised in *VAV^cre^Keap^fl/fl^* mice compared to *Keap^fl/fl^* mice, frequencies and numbers of CD3^+^CD4^+^CD25^+^Foxp3^+^ as well as CD11b^+^Gr-1^+^ cells were altered significantly. CD11b^+^Gr-1^+^ cells are already expanded in spleens of younger mice but their numbers increased progressively with age (Figure 1E) in correlation with spleen weight (Figure 1B). The dominant cell type among CD11b^+^ cells of *VAV^cre^Keap^fl/fl^* mice were Ly6G^+^ PMN-MDSCs, and levels of these were significantly enhanced (Figure 1F). It has been shown before that *Keap^fl/fl^* mice already reveal reduced expression of Keap1 protein in various tissues compared to WT mice (21), we therefore additionally compared numbers of CD11b^+^Gr-1^+^ cells in B6-WT and *Keap^fl/fl^* mice, however, numbers of MDSC were not altered in these groups (Figure S1C in Supplementary Material) and we concluded from this that *Keap^fl/fl^* and WT controls are comparably fitting controls in our experiments. NAD(P)H quinine oxidoreductase (Nqo-1), which is one of the most specific Nrf2 targets, was strongly enhanced in *VAV^cre^Keap^fl/fl^* CD11b^+^Gr-1^+^ cells compared to *Keap^fl/fl^* cells, while CD11b^+^Gr-1^+^ cells from *Nrf2*^−/−^ mice exhibited reduced Nqo-1 expression (Figure S1D in Supplementary Material), which shows that Nrf2 is hyperactivated in *VAV^cre^Keap^fl/fl^* compared to *Keap*^fl/fl^ cells. CD11b^+^Gr-1^+^ cells in BM are IMCs, which differentiate into mature granulocytes, macrophages, or DCs in healthy individuals. However, proliferation of IMCs and blockade of their differentiation can result in the accumulation of MDSCs in lymphoid organs. In most cases, accumulation of MDSCs is caused by pathogenic conditions such as cancer, inflammation, and autoimmunity (1). We therefore wanted to find out whether the observed expansion of CD11b^+^Gr-1^+^ cells is a secondary effect or whether it is indeed regulated by cell autonomous Nrf2/Keap1 signaling in these cells. To answer this question, we performed *in vitro* MDSC generation assays with BM-derived cells from *Keap^fl/fl^*, Nrf2^−/−^, and *VAV^cre^Keap^fl/fl^* mice. Culturing of BM cells from naive mice with GM-CSF and IL-6 has previously been shown to lead to an enrichment of suppressive Gr-1^+^CD11b^+^ cells (22, 23). Interestingly, Nrf2-deficient BM-derived cells exhibited a quite low expression of Gr-1 and CD11b in comparison to *Keap^fl/fl^* BM-derived cells, whereas *VAV^cre^Keap^fl/fl^* cells showed the highest capacity to acquire a CD11b^+^Gr-1^+^ phenotype (Figure 1G). To further confirm that Nrf2/Keap1 signaling directly induces CD11b^+^Gr-1^+^ cells, we generated mixed BM chimeras. To this end, we transferred equal numbers of CD45.1 WT and CD45.2 *VAV^cre^Keap^fl/fl^* BM cells into lethally irradiated *Rag*^−/−^ mice, which led to complete reconstitution of the hematopoietic system within 8 weeks. As expected, numbers of splenic CD11b^+^Gr-1^+^ CD45.1^+^ (WT derived) cells were lower than numbers of splenic CD45.2^+^ (*Keap*^−/−^ derived) cells in the recipient mice (Figures 1H,I).

### Nrf2 Activation in Myeloid Cells Results in Cells Which Display MDSC Characteristics

**Figure 2:**
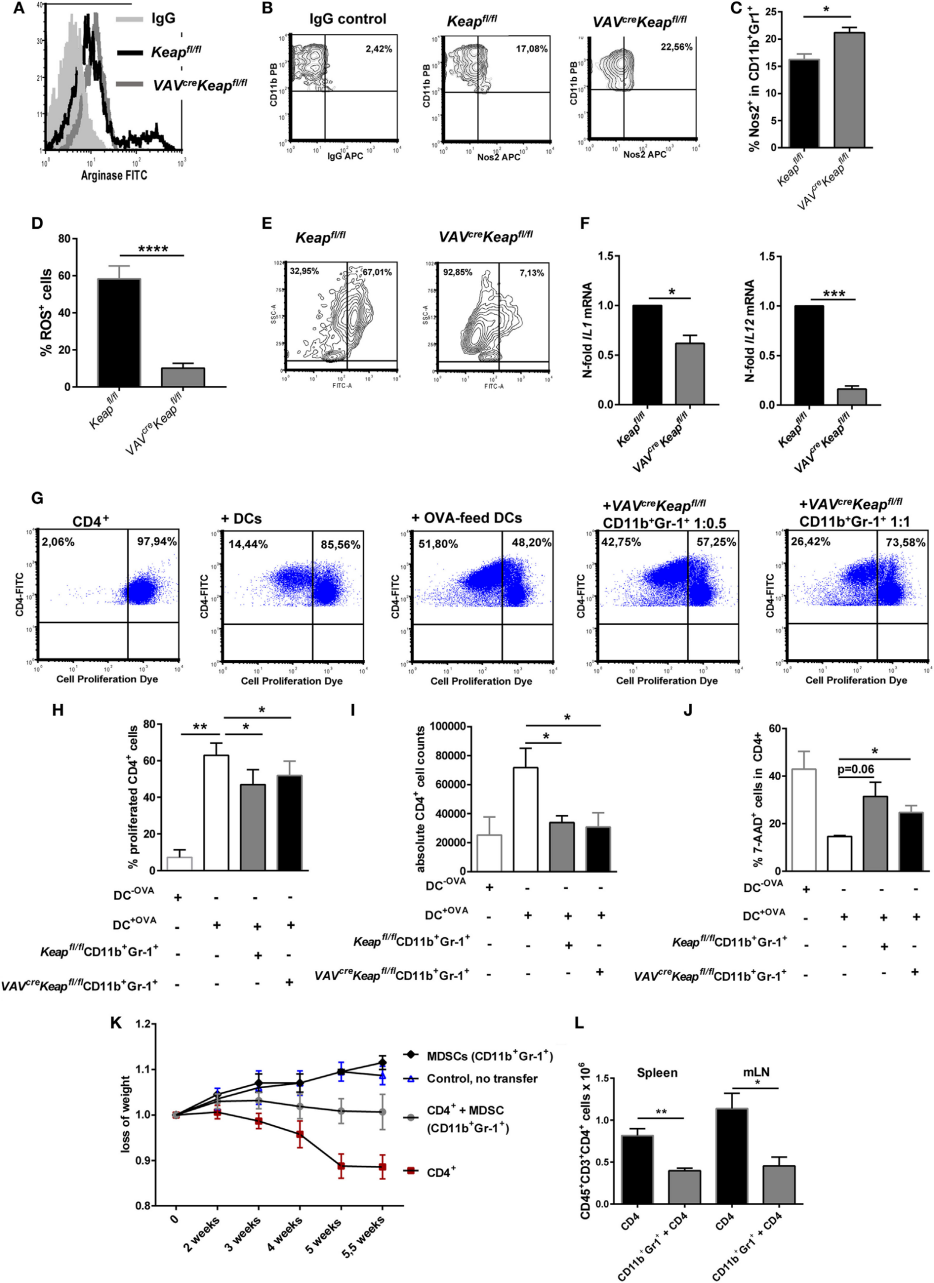
CD11b^+^Gr-1^+^ cells from *VAV^cre^Keap^fl/fl^* reveal characteristics of myeloid-derived suppressor cells (MDSCs). **(A)** Representative arginase-histogram showing overlays of pre-gated CD11b^+^Gr-1^+^ cells in spleens from *Keap^fl/fl^* (black), *VAV^cre^Keap^fl/fl^* mice (gray), and an appropriate isotype control. **(B)** Flow cytometric analysis of Nos2 expression in pre-gated CD11b^+^Gr-1^+^ cells from spleens. Representative contour plots showing isotype control (left) and Nos2 expression from *Keap^fl/fl^* (middle) and *VAV^cre^Keap^fl/fl^* mice (right). **(C)** Statistical analysis of Nos2-positive cells in pre-gated CD11b^+^Gr-1^+^ *Keap^fl/fl^* (*n* = 3) and *VAV^cre^Keap^fl/fl^* (*n* = 3) mice, two-tailed unpaired *t*-test. Bars indicate mean and error bars SEM of three mice per group. **(D)** Statistical analysis of ROS^+^ cells in pre-gated CD11b^+^Gr-1^+^ from *Keap^fl/fl^* (*n* = 6) and *VAV^cre^Keap^fl/fl^* (*n* = 8) mice, two-tailed unpaired *t*-test. **(E)** Representative contour plots showing reactive oxygen species (ROS) positive cells pre-gated on CD11b^+^Gr-1^+^ cells cultured at 37°C for 1 h. **(F)** N-fold mRNA expression of cytokines in MACS isolated *Keap^fl/fl^* and *VAV^cre^Keap^fl/fl^* CD11b^+^Gr-1^+^ (*n* = 4) cells analyzed by RT-qPCR. Bars indicate mean and error bars SEM, two-tailed one sample test. **(G–J)** OT-II CD4^+^ T cells were labeled with the cell proliferation dye eFluor 660 and cultured alone, in the presence of DCs, or in the presence of OVA-fed DCs, or co-cultured with OVA-fed DCs or different ratios of Gr1^+^CD11b^+^ cells from *VAV^cre^Keap^fl/fl^* mice. **(G)** After 3 days of culture, T cell proliferation was measured by loss of eFluor fluorescence on flow cytometry. **(H)** Statistical analysis of cell proliferation, as assessed by percentages of proliferated cells. Bars indicate the mean of three independent experiments and error bars SEM (two-tailed, paired *t*-test). **(I)** Statistical analysis of absolute CD4^+^ cells after 3 days of culture. **(J)** Statistical analysis of dead CD4^+^ T cells, as determined by incorporation of 7-AAD, two-tailed unpaired *t*-test. Bars indicate the mean of three independently performed experiments and error bars SEM. **(K)** CD4^+^CD25^−^ transfer colitis: body weight as a percent of starting weight of control mice (*n* = 4, blue symbols), MDSC recipient control mice (*n* = 2, black), CD4^+^CD25^−^ recipient mice (*n* = 8, red), and CD4^+^CD25^−^ + MDSCs recipient mice (gray, *n* = 7) over the course of 5.5 weeks. **(L)** Statistical analysis of frequencies of CD4^+^ T cells in spleen and mesenterial lymph nodes (mLNs).

Expression of Gr-1 and CD11b are known characteristics of MDSCs. However, these markers are expressed by a quite heterogeneous cell population and additional attributes are required to define cells as MDSCs. These include the expression of immune suppressive factors such as arginase (encoded by *ARG1*), inducible nitric oxide synthase (also known as Nos2) an increase in the production of ROS. Interestingly, *VAV^cre^Keap^fl/fl^* CD11b^+^ Gr-1^+^ cells showed all the characteristic hallmarks of MDSCs such as production of arginase (Figure 2A; Figure S2A in Supplementary Material) and Nos2 (Figures 2B,C). ROS production was lower in *VAV^cre^Keap^fl/fl^* CD11b^+^ Gr-1^+^ cells than in *Keap^fl/fl^* cells (Figures 2D,E). This may be explained by a high activity of the anti-oxidative machinery in *VAV^cre^Keap^fl/fl^* cells, leading to a rapid scavenging of produced ROS molecules in these cells. Interestingly, most notably *VAV^cre^Keap^fl/fl^* Ly6G^+^ CD11b^+^ cells revealed a reduction of ROS molecules (Figure S2B in Supplementary Material). In addition, such as WT CD11b^+^Gr-1^+^ cells, *VAV^cre^Keap^fl/fl^* CD11b^+^Gr-1^+^ cells showed a lower expression of maturation and differentiation markers like CD11c, CD80, CD86, and MHC-II compared to CD11b^+^Gr-1^−^ cells (Figure S2C in Supplementary Material). With regard to inflammatory cytokines, IL-6 was not detectable and levels of *IL-1* and *IL-12* were markedly reduced in *VAV^cre^Keap^fl/fl^* compared to *Keapfl/fl* CD11b^+^Gr-1^+^ cells (Figure 2F). Furthermore, inhibition of T cells by means of T cell suppression assays is the “gold” standard for evaluation of MDSC function (2). Addition of *Keapfl/fl* and *VAV^cre^Keap^fl/fl^* CD11b^+^Gr-1^+^ cells to antigen-specific stimulated T cells reduced percentages of proliferated cells (Figures 2G,H), diminished absolute numbers of T cells (Figure 2I), and enhanced percentages of dead T cells (Figure 2J). Furthermore, *VAV^cre^Keap^fl/fl^* CD11b^+^Gr-1^+^ suppressed T cell mediated transfer colitis (Figure 2K). While RAG-deficient (RAG2^−/−^) recipients of CD4^+^ T cells suffered from severe colitis with weight loss and high-grade intestinal inflammation (Figure 2K; Figures S3A,B,C in Supplementary Material), co-transfer of Keap-deficient CD11b^+^Gr-1^+^ cells markedly reduced loss of weight and intestinal inflammation (Figure 2K; Figures S3B,C in Supplementary Material). Absolute numbers of CD3^+^CD4^+^ cells in spleens and mLNs were reduced as well (Figure 2L). Furthermore, T_reg_ cells numbers increased (Figure S3D in Supplementary Material) while levels of inflammatory cytokines in the gut were markedly reduced in RAG2^−/−^ mice transferred with MDSCs in addition to CD4^+^ T cells (Figure S3E in Supplementary Material).

We conclude from these experiments that CD11b^+^Gr-1^+^ *VAV^cre^Keap^fl/fl^* cells show all hallmarks and functional properties of MDSCs, such as production of arginase and Nos2 but low expression of maturation and differentiation markers and inflammatory cytokines and a high ability to suppress T cell proliferation *in vitro* and *in vivo*.

### Metabolic Pathways and Cell Cycle Pathways Are Enriched in MDSC With Constitutive Nrf2 Activation

**Figure 3:**
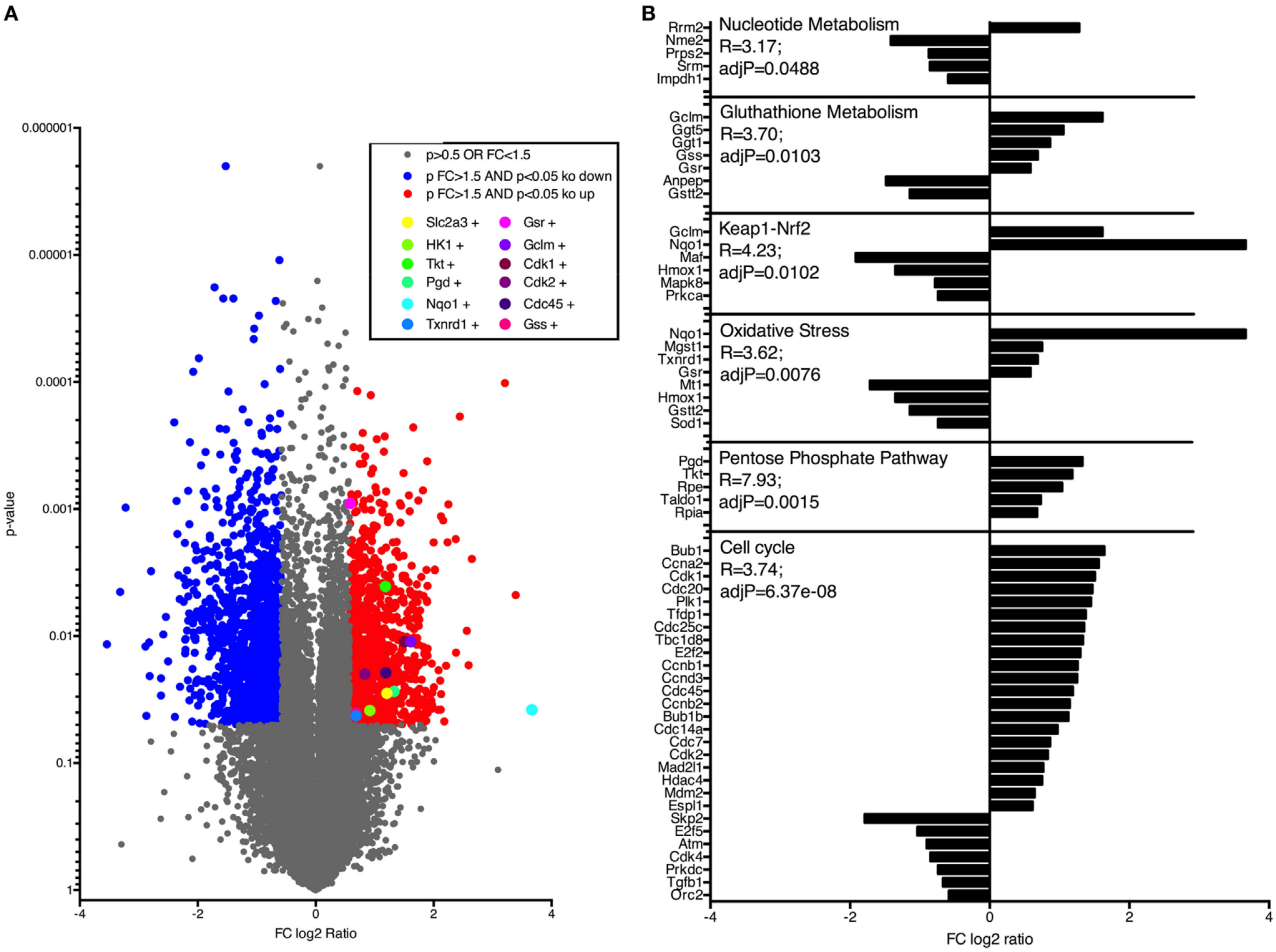
Nrf2 activates genes regulating cell cycle and metabolic pathways in myeloid-derived suppressor cells (MDSCs). **(A)** Gene expression in CD11b^+^Gr-1^+^ cells from *Keap^fl/fl^* and *VAV^cre^Keap^fl/fl^* mice. Colors indicate significant upregulation (of at least 1.5-fold; red) or downregulation (of at least 1.5-fold; blue). **(B)** Selection of pathways and associated genes which were significantly enriched.

Next, to investigate how Nrf2/Keap1 signaling induces expansion of MDSCs, we performed whole transcriptome analysis in MDSCs isolated from *VAV^cre^Keap^fl/fl^* mice and *Keap^fl/fl^* mice using Affymetrix arrays. Several genes, which belong to the *oxidative stress pathway, Nrf2/Keap1 signaling pathway*, and *glutathione metabolism*, were activated in *VAV^cre^Keap^fl/fl^* MDSCs and differed significantly from WT MDSCs, which confirm constitutive Nrf2 activation in these cells at a transcriptional level (Figures 3A,B). Most interestingly, in addition to this, we noticed altered expression of genes belonging to metabolic as well as cell cycle pathways (Figures 3A,B). In detail, genes of the *cell cycle*, the *pentose phosphate pathway* (*PPP*), and *nucleotide, metabolism* showed enhanced expression in *VAV^cre^Keap^fl/fl^* compared to WT MDSCs.

### Nrf2 Enhances Proliferation of CD11b^+^Gr-1^+^ Cells

**Figure 4:**
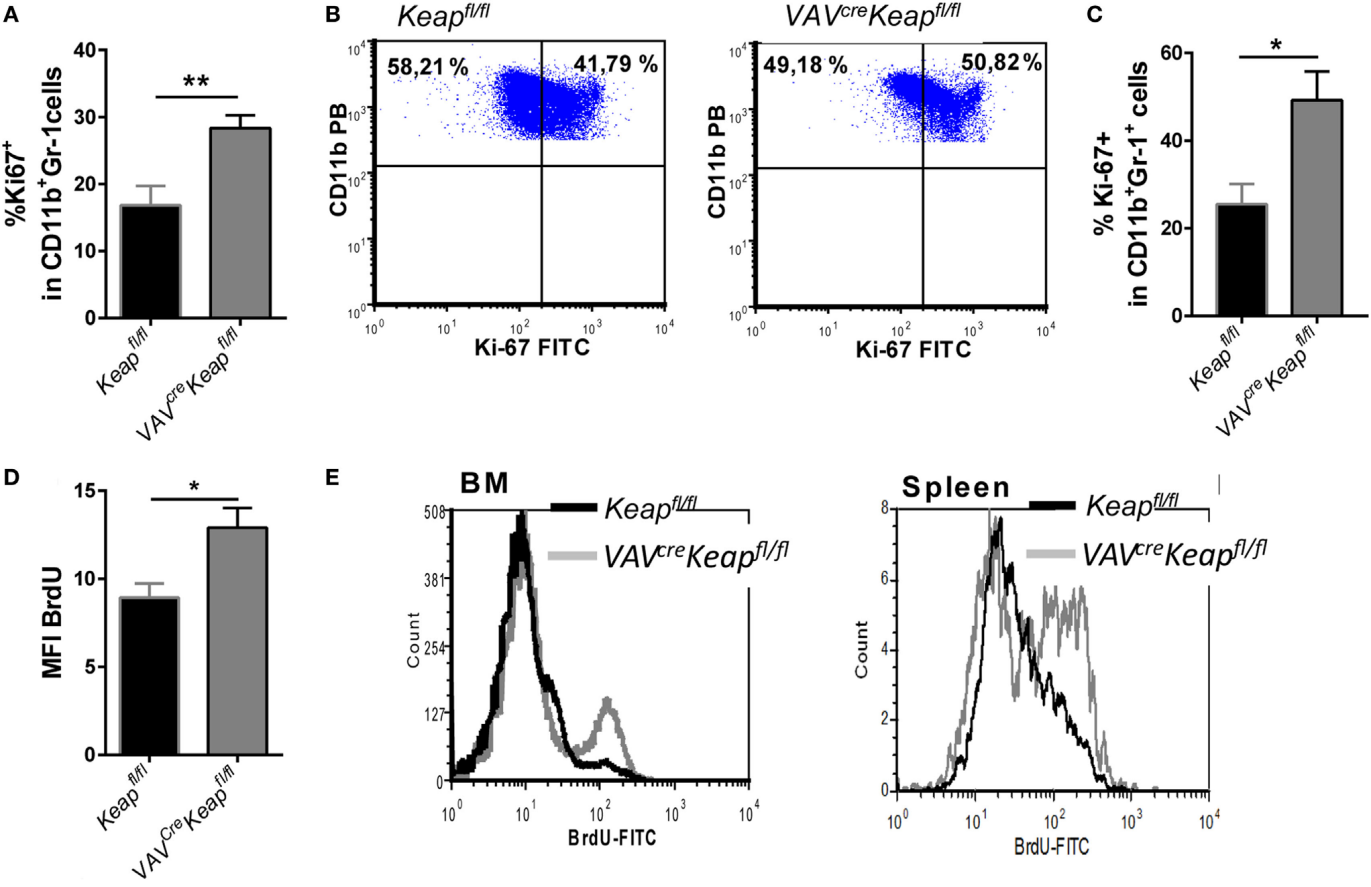
Nrf2 induces proliferation of myeloid-derived suppressor cells (MDSCs). **(A)** Statistical analysis of Ki-67 expression in pre-gated splenic CD11b^+^Gr-1^+^ *Keap^fl/fl^* (*n* = 5) and *VAV^cre^Keap^fl/fl^* (*n* = 5) cells. Bars indicate mean of mean fluorescence intensity and error bars SEM. **(B)** Representative dot plots showing Ki-67 expression in pre-gated CD11b^+^Gr-1^+^ BM-derived cells. **(C)** BM-derived cells were incubated with GM-CSF and IL-6 for 8 days and frequencies of Ki-67^+^ cells within CD11b^+^Gr-1^+^ cells were assessed by flow cytometry. Bars indicate mean ± SEM of four independently performed experiments. **(D)** *Keap^fl/fl^* and *VAV^cre^Keap^fl/fl^* mice were fed orally with BrdU for 14 days. Statistical analysis of BrdU incorporation among pre-gated CD11b^+^Gr-1 cells of *Keap^fl/fl^* (*n* = 5) and *VAV^cre^Keap^fl/fl^* (*n* = 6) mice. **(E)** Representative histogram shows overlays of BrdU incorporation in pre-gated CD11b^+^Gr-1^+^ cells of spleen and BM. Two-tailed unpaired *t*-tests were used to determine *p*-values for all statistical analysis.

Based on our microarray data, we hypothesized that the accumulation of MDSCs in spleen is caused by a higher proliferation rate. Analysis of Ki-67 expression in the spleen and BM cells confirmed a larger growth fraction within *VAV^cre^Keap^fl/f^*^l^ CD11b^+^Gr-1^+^ cells compared to *Keap^fl/fl^* cells (Figures 4A,B). Furthermore, *in vitro* generated MDSCs from *VAV^cre^Keap^fl/f^*^l^ mice displayed higher Ki-67 expression as well (Figure 4C). In addition, BM CD11b^+^Gr-1^+^ cells from *VAV^cre^Keap^fl/fl^* mice exhibited a higher BrdU incorporation than the respective CD11b^+^Gr-1^+^ cells from *Keap^fl/fl^* (Figures 4D,E) while BrdU incorporation into CD11b^+^Gr1^+^ cells from *VAV^cre^Keap^fl/f^*^l^ spleens was only tendentially increased (Figure 4E). However, the rates of apoptosis, as analyzed by the frequencies of early apoptotic (AnnexinV^+^ cell viability dye^−^) and late apoptotic (AnnexinV^+^ cell viability dye^+^) MDSCs were the same in *Keap^fl/fl^* and *VAV^cre^Keap^fl/fl^* mice (Figure S4 in Supplementary Material). From that, we conclude that Nrf2 accelerates proliferation of MDSCs without affecting apoptosis.

### Nrf2 Enhances Metabolic Activity of CD11b^+^Gr-1^+^Cells

**Figure 5:**
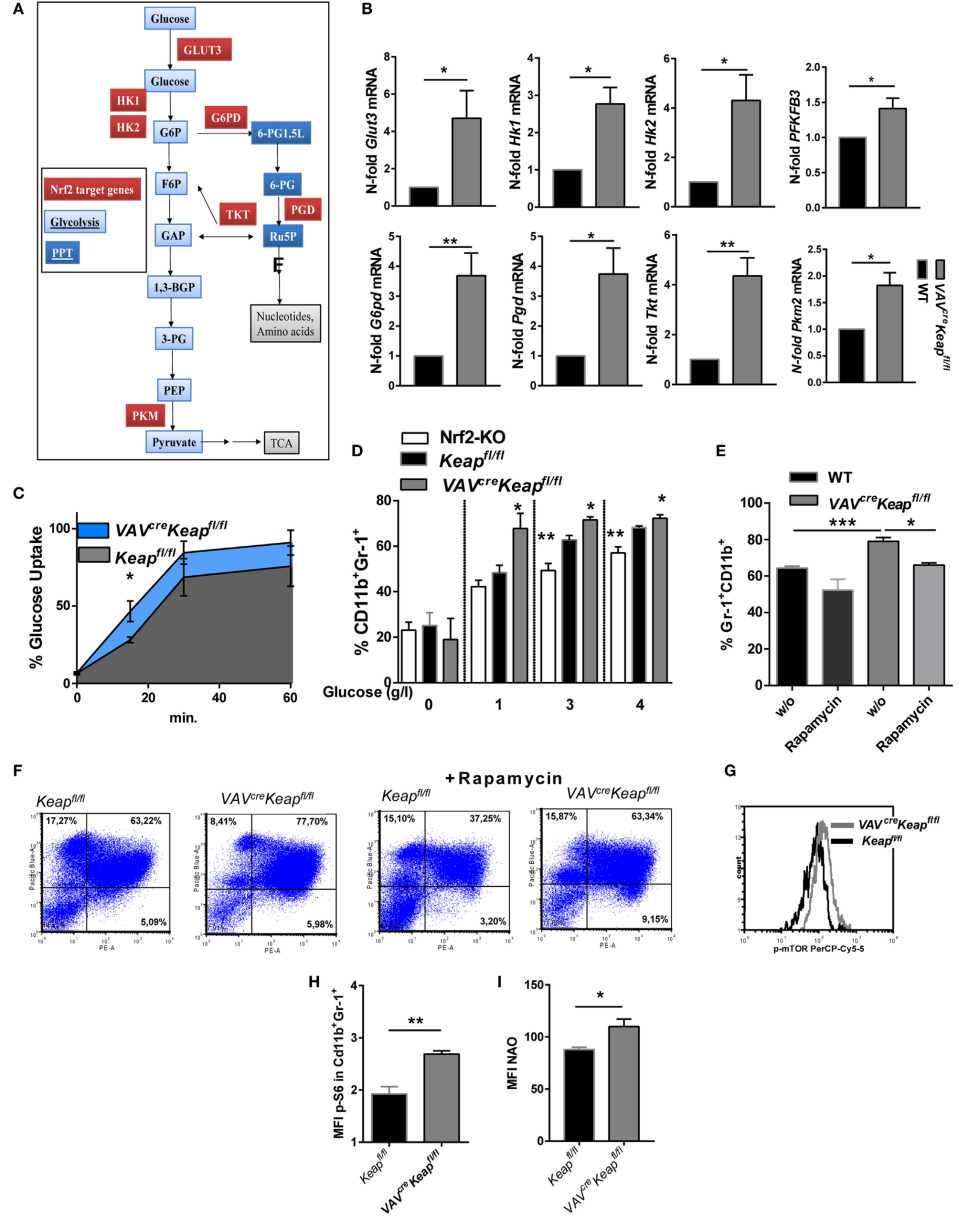

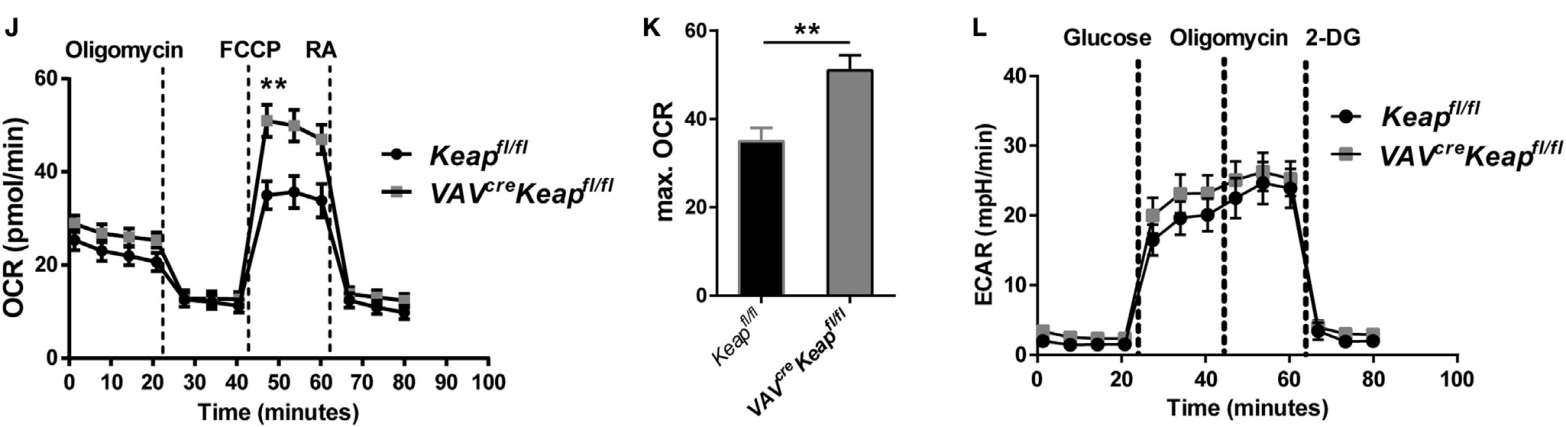
Nrf2 induces metabolic activity in myeloid-derived suppressor cells (MDSCs). **(A)** Metabolic enzymes regulated by Nrf2 in glucose metabolism in MDSCs (own microarray and RT-qPCR data). **(B)** N-fold mRNA expression of metabolic enzymes in MACS isolated *Keap^fl/fl^* and *VAV^cre^Keap^fl/fl^* CD11b^+^Gr-1^+^ (*n* = 7–8, PFKFB3 *n* = 5) cells analyzed by RT-qPCR. Bars indicate mean and error bars SEM, two-tailed one sample test. **(C)** 2NBD-glucose incorporation was analyzed in CD11b^+^Gr1^+^ splenocytes from *Keap^fl/fl^* (*n* = 3) and *VAV^cre^Keap^fl/fl^* (*n* = 4) mice at different time-points, two-tailed unpaired *t*-test. **(D)** BM-derived cells were incubated with GM-CSF and IL-6 for 8 days with different glucose concentrations, and frequencies of CD11b^+^Gr-1^+^ cells were assessed by flow cytometry. Bars indicate mean ± SEM of three independent experiments per group, two-tailed unpaired *t*-test. **(E,F)** BM-derived cells were incubated with GM-CSF and IL-6 for 8 days in the absence or presence of 1 µM rapamycin, *n* = 4, two-tailed unpaired *t*-test. **(G)** Representative histogram of five experiments (*p* = 0.0155, two-tailed paired *t*-test) showing p-mTOR expression in splenic CD11b^+^Gr-1^+^ cells. **(H)** Mean fluorescent intensity of pS6 expression in CD11b^+^Gr-1^+^ cells. **(I)** Statistical analysis of mitochondrial mass of *Keap^fl/fl^* (*n* = 4) and *VAV^cre^Keap^fl/fl^* (*n* = 4) CD11b^+^Gr-1^+^ cells evaluated by assessing NAO mean fluorescence intensity, *n* = 3, two-tailed unpaired *t*-test. **(J)** OCR measured under basal conditions and after addition of the indicated drugs. Points indicate mean from six *Keap^fl/fl^* (*n* = 6) and six *VAV^cre^Keap^fl/fl^* (*n* = 6) CD11b^+^Gr-1^+^ cells, error bars SEM. **(K)** Statistical analysis of max. OCR. Bars indicate mean ± SEM. **(L)** ECAR measured under basal conditions and after addition of the indicated drugs. Points indicate mean from six *Keap^fl/fl^* (*n* = 6) and six *VAV^cre^Keap^fl/fl^* (*n* = 6) CD11b^+^Gr-1^+^ cells, error bars SEM, two-tailed unpaired *t*-test.

The metabolic characteristics regulating MDSCs have not yet been fully elucidated and may also differ within this quite heterogeneous cell population. Tumor-infiltrating MDSCs increase fatty acid oxidation compared to splenic MDSCs (24). On the other hand, rapamycin, the specific inhibitor of mTOR, decreased M-MDSC in mice with allografts or tumors (25). A significant enrichment of genes of the *PPP* was observed in our microarray data, together with an enhancement of genes involved in glycolysis. RT-qPCR was used to validate upregulation of the following genes: *glucose transporter 3* (*Glut3, SLC2A3*), the glucose receptor of white blood cells, *hexokinase (Hk)1* and *Hk2*, enzymes responsible for committing glucose to the glycolytic pathway, *6-phosphofructo-2-kinase/fructose-2,6-biphosphatase 3* (PFKFB3) which is known as vital regulator of glycolysis and furthermore promotes cell cycle progression, *glucose-6-phosphate dehydrogenase* (*G6pd*) the rate-limiting enzyme of the *PPP, phosphogluconate dehydrogenase* (*Pgd*), the second dehydrogenase in the *PPP, transketolase* (*Tkt*), which delivers excess sugar phosphates for glycolysis in the *PPP*, and *pyruvate kinase isozyme M2* (*Pkm*2), which catalyzes the last step within glycolysis (Figures 5A,B). In addition, glucose uptake, as measured by flow cytometry, was faster in *VAV^cre^Keap^fl/fl^* MDSCs compared to *Keap^fl/fl^* MDSCs (Figure 5C) and glucose availability was a prerequisite for generation of MDSCs *in vitro* (Figure 5D). MDSC generation in WT cells was enhanced by glucose in a dose-dependent manner, while *VAV^cre^Keap^fl/fl^* cells differentiated into MDSCs even with low amounts of glucose, which suggests a more efficient uptake and faster utilization of glucose. Nrf2-deficient cells benefit from higher glucose levels, but failed to reach the same frequencies as *Keap^fl/fl^* cells even at high glucose concentrations (Figure 5D). Moreover, rapamycin, a specific mTOR inhibitor which is known to decrease glucose uptake during MDSC differentiation *in vitro* and thereby inhibits MDSC differentiation *in vitro* (25) restores the enhanced MDSC differentiation of *VAV^cre^Keap^fl/f^*^l^ BM cells to WT levels (Figures 5E,F). P-mTOR expression was enhanced in splenic *VAV^cre^Keap^fl/fl^* MDSCs as well (Figure 5G) and activation of mTOR signaling was reflected in the phosphorylation of S6 ribosomal protein (p-S6) (Figure 5H). In addition, mitochondrial mass was enhanced in *VAV^cre^Keap^fl/fl^* MDSCs (Figure 5I). Seahorse assays revealed higher maximal respiration rates (OCR) (Figures 5J,K), while extracellular acidification rates (ECAR), a measurement of lactate production, were not enhanced (Figure 5L), which in addition to the higher mitochondrial mass rates might suggests that *VAV^cre^Keap^fl/fl^* MDSCs use oxidative phosphorylation to generate ATP from glucose instead of glycolysis with subsequent lactate secretion. Overall, we conclude from these data that Keap1-deficient MDSCs exhibit higher uptake of abundant nutrients and a higher overall metabolic activity, which leads to an increase in metabolic pathways and pushes them into proliferative states.

### Nrf2 Activation Resembles LPS-Induced MDSC Expansion

**Figure 6:**
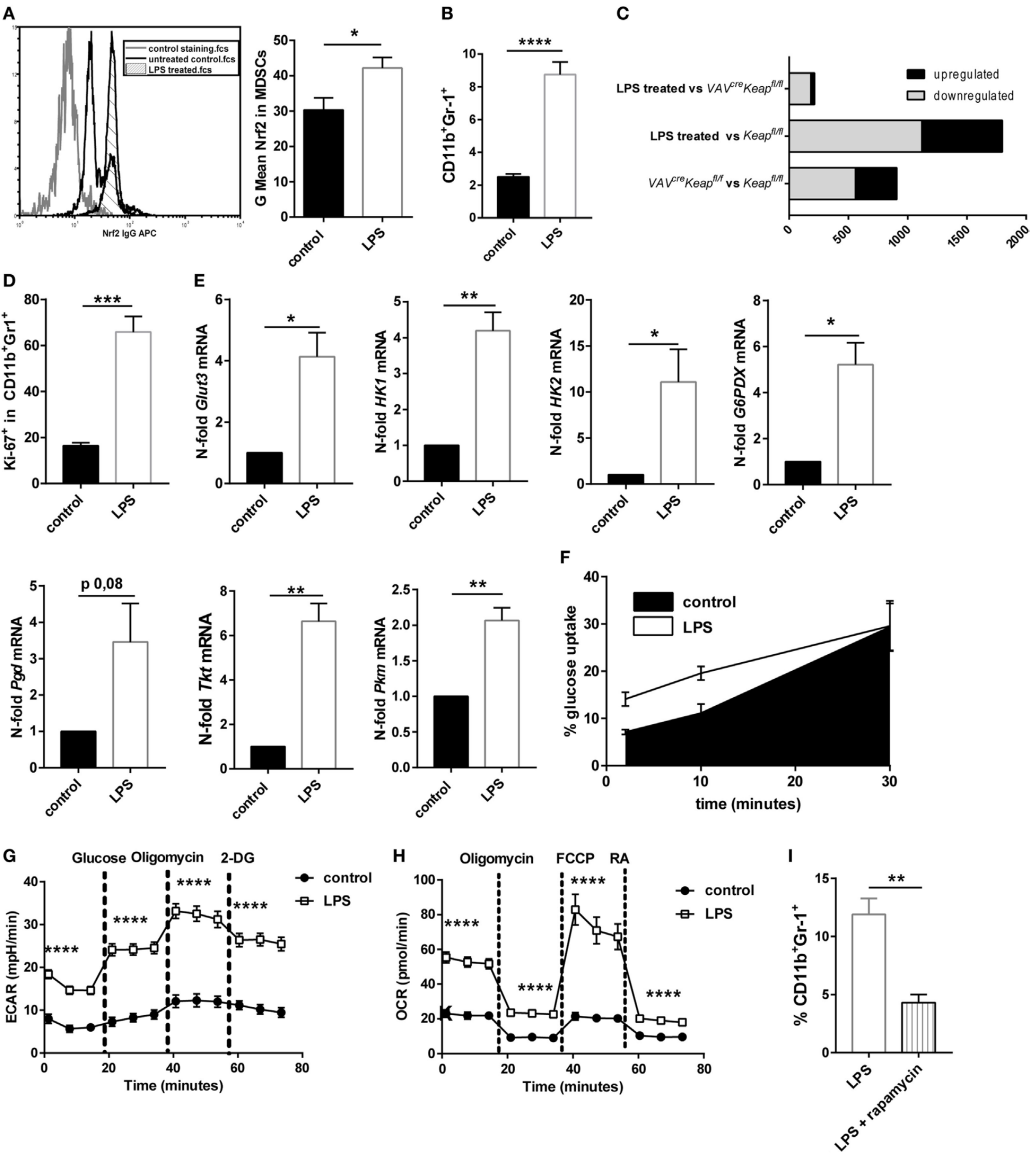
Nrf2 activation resembles LPS-induced myeloid-derived suppressor cell (MDSC) expansion. **(A)** MFI of Nrf2 expression in CD11b^+^Gr-1^+^ cells of untreated mice and mice after low-dose LPS treatment, unpaired one-tailed *t*-test, *n* = 3. Bars indicate mean ± SEM. **(B)** Flow cytometric analysis of CD11b^+^Gr-1^+^ cells in spleens from untreated *Keap^fl/fl^* mice and mice after LPS treatment. Bars indicate mean and error bars SEM of three experiments with a total of nine mice per group. **(C)** Diagram of differentially expressed genes (*p* < 0.05, >2-fold) between LPS-induced MDSCs vs. *VAV^cre^Keap^fl/fl^* (top), LPS-induced MDSCs vs. *Keap^fl/fl^* (middle), and of *VAV^cre^Keap^fl/fl^* and WT (bottom). **(D)** Statistical analysis of Ki-67 expression in pre-gated splenic CD11b^+^Gr-1^+^ cells from untreated (*n* = 4) and LPS treated (*n* = 4) mice. Bars indicate mean of mean fluorescence intensity and error bars SEM, two-tailed, unpaired *t*-test. **(E)** N-fold mRNA expression of metabolic enzymes in MACS isolated CD11b^+^Gr-1^+^ cells analyzed by RT-qPCR. Bars indicate mean and error bars SEM, *n* = 5, two-tailed, one sample test. **(F)** 2NBD-glucose incorporation was analyzed in CD11b^+^Gr1^+^ splenocytes from control (*n* = 6) and LPS treated *Keap^fl/fl^* mice (*n* = 6) mice at different time-points. **(G)** ECAR measured under basal conditions and after addition of the indicated drugs. Points indicate mean from three control and three LPS treated *Keap^fl/fl^* mice ± SEM of quintuplicates. **(H)** OCR measured under basal conditions and after addition of the indicated drugs. Points indicate mean CD11b^+^Gr-1^+^ cells from three control and three LPS treated *Keap^fl/fl^* mice ± SEM of quintuplicates. **(I)** Flow cytometric analysis of CD11b^+^Gr-1^+^ cells in spleens from *Keap^fl/fl^* mice 4 days after LPS treatment and daily administration of vehicle or rapamycin. Bars indicate mean and error bars SEM of three experiments with a total of four mice per group.

Myeloid-derived suppressor cells strongly expand under septic conditions in mice and men (3, 4). We also observed higher levels of Nrf2 protein expression in CD11b^+^Gr-1^+^ cells after treating mice with sublethal doses LPS (Figure 6A). We therefore speculated whether LPS-induced MDSCs are regulated by Nrf2 signaling and would show similarities with MDSCs of *VAV^cre^Keap^fl/fl^* mice. Mice were treated with sublethal doses of LPS (5 mg/kg/bw) which resulted in a significant enrichment of CD11b^+^Gr-1^+^ cells in spleens (Figure 6B). Systems biology analysis identified a high number of alike regulated genes in LPS-treated MDSCs and *VAV^cre^Keap^fl/fl^* MDSCs (Figure 6C), which revealed strikingly more transcriptional similarities (e.g., less differentially expressed genes) between LPS-induced MDSCs and *Keap^fl/fl^* MDSCs than between MDSCs of *Keap^fl/fl^* mice vs. *Keap^fl/fl^* mice (Figure 6C). In detail, we identified 1,798 genes showing significant expression changes (>2-fold change, *p* < 0.05) in LPS-treated vs. *Keap^fl/fl^* mice. By contrast, only 214 genes were differentially expressed between LPS treated and *VAV^cre^Keap^fl/fl^* MDSCs using the same significance criteria (Figure 6C). In addition, pathway gene set enrichment analysis revealed similar patterns in *VAV^cre^Keap^fl/fl^* MDSC and LPS-induced MDSCs, which included metabolic pathways like the *PPP* pathway, as well as *nucleotide metabolism* and the *cell cycle* pathway. The same was true for the statin pathway, *complement activation* and *macrophage markers* (Figure S5 in Supplementary Material). Consequently, LPS-induced CD11b^+^Gr-1^+^ cells revealed higher Ki-67 expression (Figure 6D) and enhanced mRNA levels of genes, which belong to the glucose and PPP pathway (Figure 6E) as well as faster glucose uptake (Figure 6F). LPS-induced CD11b^+^Gr-1^+^ cells had increased ECAR on a basal level and after addition of glucose and furthermore after addition of Oligomycin, which blocks mitochondrial ATP production and promotes maximal rates of glycolysis (Figure 6G). In addition to this, OCR was enhanced as well under basal conditions but also after addition of Oligomycin and FCCP, which uncouples oxidative phosphorylation from electron transport and allows maximal respiration (Figure 6H). While OCR was enhanced in *VAV^cre^Keap^fl/fl^* MDSCs as well, an enhanced ECAR seems to be more specific for LPS-induced MDSCs and might occur independently of Nrf2 signaling. To proof if inhibition of mTOR influences LPS-mediated induction of MDSC *in vivo*, we treated mice with sublethal doses of LPS together with rapamycin. *In vivo* administration of rapamycin (2 mg/kg/bw i.p. every day) significantly reduced numbers of CD11b^+^Gr-1^+^ cells in spleens of LPS-treated mice (Figure 6I). In conclusion, these data show that LPS-induced MDSCs show some similarities with Nrf2-activated MDSCs and are as well characterized by an activation of metabolic pathways and higher proliferation states.

### Nrf2 Activation Contributes to TLR4-Mediated MDSCs Expansion

**Figure 7:**
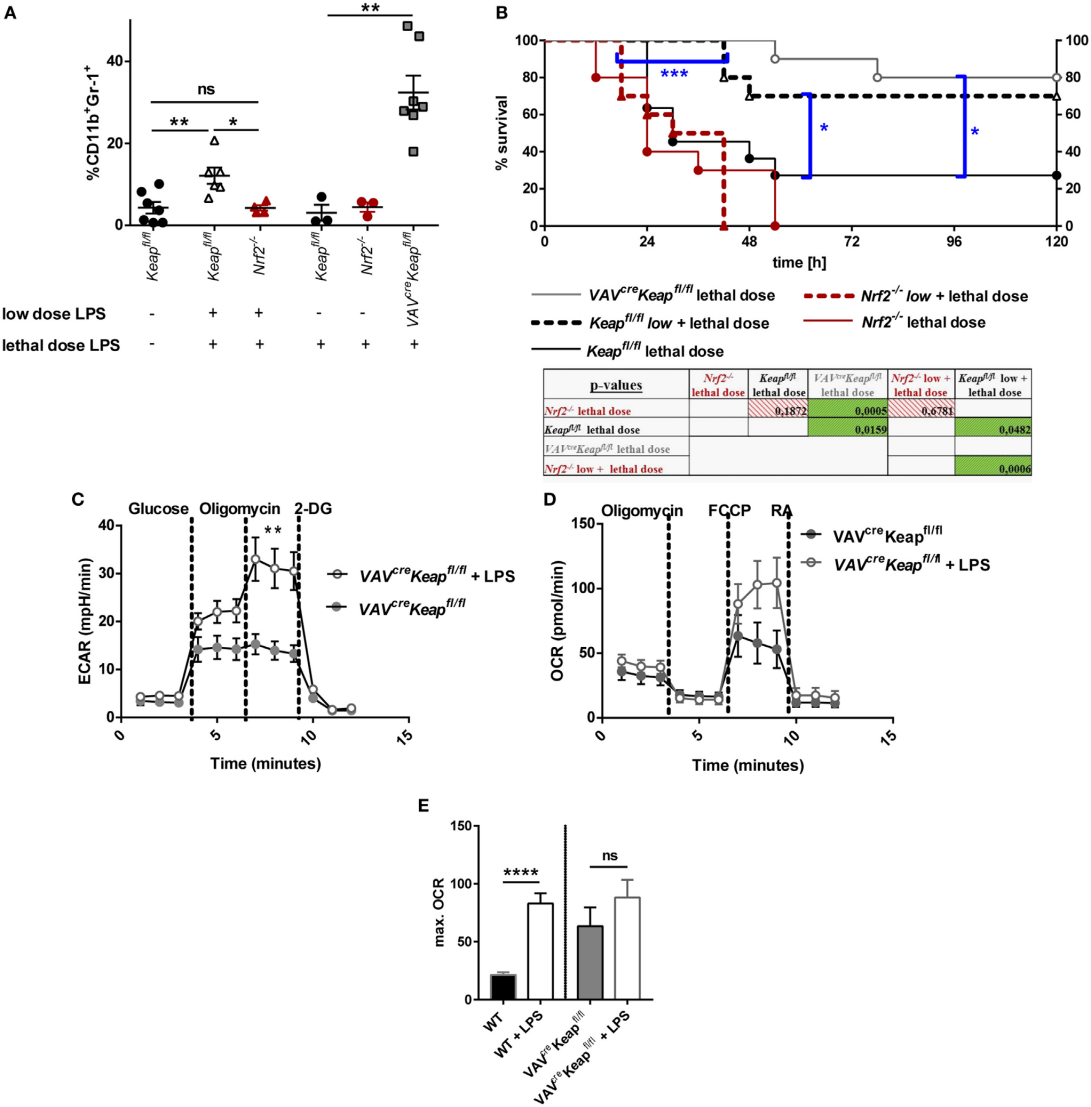
Nrf2 activation in myeloid-derived suppressor cells (MDSCs) regulates LPS-mediated disease. **(A)** Mice were injected with either a low dose of LPS (5 mg/kg of body weight) and subsequently with a lethal dose of LPS (30 mg/kg of body weight) or solely with a lethal dose of LPS (30 mg/kg of body weight). Mice were euthanized depending on a scoring system (usually within the first 48 h after the single lethal dose) or 72 h after injection of the subsequent lethal dose and frequencies of CD11b^+^Gr-1^+^ cells were determined, unpaired, two-tailed *t*-test, ±SEM, *N* = 10 mice/group. **(B)** Kaplan–Meyer survival curves of mice, *p*-values were determined by Log-rank/Mantel-Cox Test of survival curves (single comparisons) and a subsequent FDR correction of single *p*-value for multiple comparison test. **(C)** ECAR measured under basal conditions and after addition of the indicated drugs. Points indicate mean from three control and three LPS treated *VAV^cre^Keap^fl/fl^* splenic MDSCs ± SEM. **(D)** OCR measured under basal conditions and after addition of the indicated drugs. Points indicate CD11b^+^Gr-1^+^ cells from three control and three LPS treated mice ± SEM. **(E)** Statistical analysis of max. OCR. Bars indicate mean ± SEM.

To finally analyze the functional significance of our findings *in vivo* we performed an acute lethal model and a tolerance sepsis model. Mice were treated with either lethal doses (acute, lethal model) or either with sublethal (tolerizing) and subsequent lethal LPS doses (tolerance model). While treatment with low doses and subsequent lethal doses of LPS expectedly induced MDSC expression in wild-type mice and induced a protection against the lethal dose, *Nrf2*^−/−^ mice had to be taken out of the experiment and sacrificed at day 2 or 3 without acquiring an enhanced MDSC population (Figures 7A,B). But it should be considered that the reduced numbers of MDSCs during the sepsis experiment in Nrf2^−/−^ mice might be related to the early death of the mice. However, mice with deletion of Nrf2 were not protected by a tolerizing dose of LPS and died after the second lethal dose of LPS, while WT mice that underwent the same procedure were protected and displayed expanded numbers of MDSCs. In addition, mice with a constitutive expression of Nrf2 in hematopoietic cells (*VAV^cre^Keap^fl/fl^*) were resistant against lethal doses of LPS even without previous treatment with tolerizing LPS doses (Figures 7A,B). To further test if LPS mediates MDSC expansion and metabolic activation by promoting Nrf2 activation, we analyzed *VAV^cre^Keap^fl/fl^* CD11b^+^Gr-1^+^ cells after tolerizing LPS treatment. While LPS significantly enhanced ECAR also in *VAV^cre^Keap^fl/fl^* CD11b^+^Gr-1^+^ cells, which was comparable to the effect in wild-type MDSCs (Figure 7C), the OCR was only slightly enhanced compared to LPS-induced OCR activation of WT CD11b^+^Gr-1^+^ cells (Figures 7D,E). This experiment shows clearly that LPS favors aerobic glycolysis and lactate production in CD11b^+^Gr-1^+^ cells independently from Nrf2, but that Nrf2 translocation indeed contributes to enhanced ATP generation from oxidative phosphorylation in MDSCs, which might be protective in LPS-induced septic shock.

## Discussion

Nrf2 is a key transcriptional regulator, driving antioxidant gene expression and protection from oxidant injury, and is activated by ROS. Nrf2 regulated genes include a battery of antioxidant enzymes such as Nqo-1. Under quiescent conditions, Nrf2 is bound to Keap1 in the cytoplasm, resulting in proteasomal degradation. Cellular stimuli, such as oxidative stress, induce conformational changes in Keap1 resulting in the release of Nrf2 (26). Subsequently, Nrf2 translocates to the nucleus and transactivates expression of genes containing an antioxidant response element in their promoter regions (27). Nrf2 has been described before as a positive regulator of myeloid differentiation (28) and it skews the differentiation potential of HSCs toward the granulocyte-monocyte lineage (29). Nevertheless, the role for Nrf2 activation in MDSCs in different relevant diseases like cancer is contra dictionary (13, 30, 31). We therefore performed a comprehensive approach to study Nrf2/Keap1 signaling in MDSCs in steady state and sepsis and identified Nrf2 as a key metabolic regulator of these immunosuppressive cells.

In detail, we could show that Nrf2/Keap1 signaling enhances MDSCs in a cell intrinsic manner, Nrf2 activated MDSCs are suppressive *in vitro* and *in vivo* and reveal other characteristics of MDSCs such as expression of arginase and NOS2 and low expression of inflammatory cytokines. Furthermore, Nrf2 induces transcriptional reprogramming of MDSCs, which resembles the transcriptional profile of LPS-induced MDSCs and might thus critically contribute to LPS-mediated tolerance. While it was shown before that Nrf2 is protective in models of sepsis by suppressing LPS-induced inflammatory cytokine expression in macrophages (32–35), these studies did not address MDSCs and our observations in metabolic reprogramming of MDSCs might critically contribute to the protective role of Nrf2 in this context.

Nrf2 is involved in metabolic reprogramming of cancer cells and in regulation of mitochondrial respiration (36–38). Furthermore, a direct mTOR activation by Nrf2 has been shown in human cell lines (39). These earlier data from other cell types support our study, as we also detected an higher metabolic activity, higher glucose uptake and mitochondrial masses, and high mTOR phosphorylation in Nrf2-induced MDSCs cells and could even block Nrf2-induced MDSC generation with rapamycin.

Collectively our study suggests that Nrf2 is a key modulator of MDSCs which might contribute to innate memory in sepsis. Nrf2 activation induces expansion of MDSCs; Nrf2 is also necessary to expand MDSCs in the situation of LPS tolerance. Finally, these MDSCs are protective in acute LPS-induced sepsis. While Nrf2 activated MDSCs share several transcriptional similarities with LPS-tolerized WT MDSCs, we found one striking difference in energy consumption between *VAV^cre^Keap^fl/fl^* MDSCs and LPS-tolerized WT MDSCs. The latter ones prefer aerobic glycolysis for ATP generation. Even the *VAV^cre^Keap^fl/fl^* MDSCs can change their metabolic expenditures after LPS treatment to enhanced aerobic glycolysis, which suggests that glycolysis with subsequent lactate production is mainly regulated independently of Nrf2. One advantage of glycolysis in comparison to oxidative phosphorylation is a better maintenance of the redox balance. Most of cellular ROS is produced during oxidative phosphorylation in the mitochondria (40). *VAV^cre^Keap^fl/fl^* MDSCs show low levels of intracellular ROS despite enhanced mitochondrial mass. This can be explained by constitutive activation and availability of antioxidant enzymes in these cells and might be an important mechanism which enables the cells to enhance mitochondrial ATP production by counteracting subsequent high ROS levels at the same time. With this regard, it is interesting that Nrf2 activation in particular enhances PMN-MDSCs, which are known to produce excessive amount of ROS (41) and that Nrf2 activation also mainly reduces this high ROS levels in PMN-MDSCs and not in M-MDSCs. Therefore, PM-MDSCs may benefit more from Nrf2 activation and subsequent reduction of oxidative stress.

Host defense against recurrent infections is mediated by innate immune memory. The phenomena of trained immunity and endotoxin tolerance are examples of such innate-type memory, with trained immunity describing an adaption that results in the long-lasting capacity to respond more strongly and tolerance describing a hypoinflammatory state. However, it is not clear if these are two fundamentally divergent programs or just represent different facets of innate memory (5). Whereas the priming with β-glucan from *Candida albicans* leads to a state of trained immunity with a potentiation of inflammatory cytokine production, TLR4 stimulation with LPS can induce a state of endotoxin tolerance and suppression of inflammatory cytokines. Recent studies showed that aerobic glycolysis is the metabolic basis for trained immunity (42). The metabolism of tolerant myeloid cells, especially of MDSCs, is less clear and was matter of our study. It is generally accepted that, naïve or tolerant cells rely mainly on oxidative phosphorylation as energy sources while activated cells, e.g., after LPS stimulation, shift their metabolism toward aerobic glycolysis (43). By contrast, leukocytes from patients with severe sepsis and immunoparalysis display a generalized metabolic defect at the level of both glycolysis and oxidative metabolism in cellular energy metabolism (5), which means that a complete metabolic reprogramming occurs between acute sepsis and immunoparalysis. We could show that tolerizing mice with a low-dose LPS induced an activation of both glycolysis and OXPHOS in MDSCs. Mice with a constitutive Nrf2 activation already revealed at least higher OCR level and were even protected without any tolerizing pretreatment. Interestingly, MDSCs generated during infection show a strong anti-inflammatory phenotype, compared to splenic CD11b^+^Gr1^+^ cells under steady-state conditions (11) and we also detected reduced level of inflammatory cytokines *in VAV^cre^Keap^fl/fl^* mice compared to untreated MDSCs. This further suggests that MDSCs need to be primed to acquire an anti-inflammatory phenotype either by LPS and/or by Nrf2 activation.

One limitation of our study is that we so far only used LPS injections as a model for sepsis, therefore further studies will also include the cecal ligation and puncture model in order to test the effects of Nrf2 activation in different animal models of sepsis.

**Figure 8:**
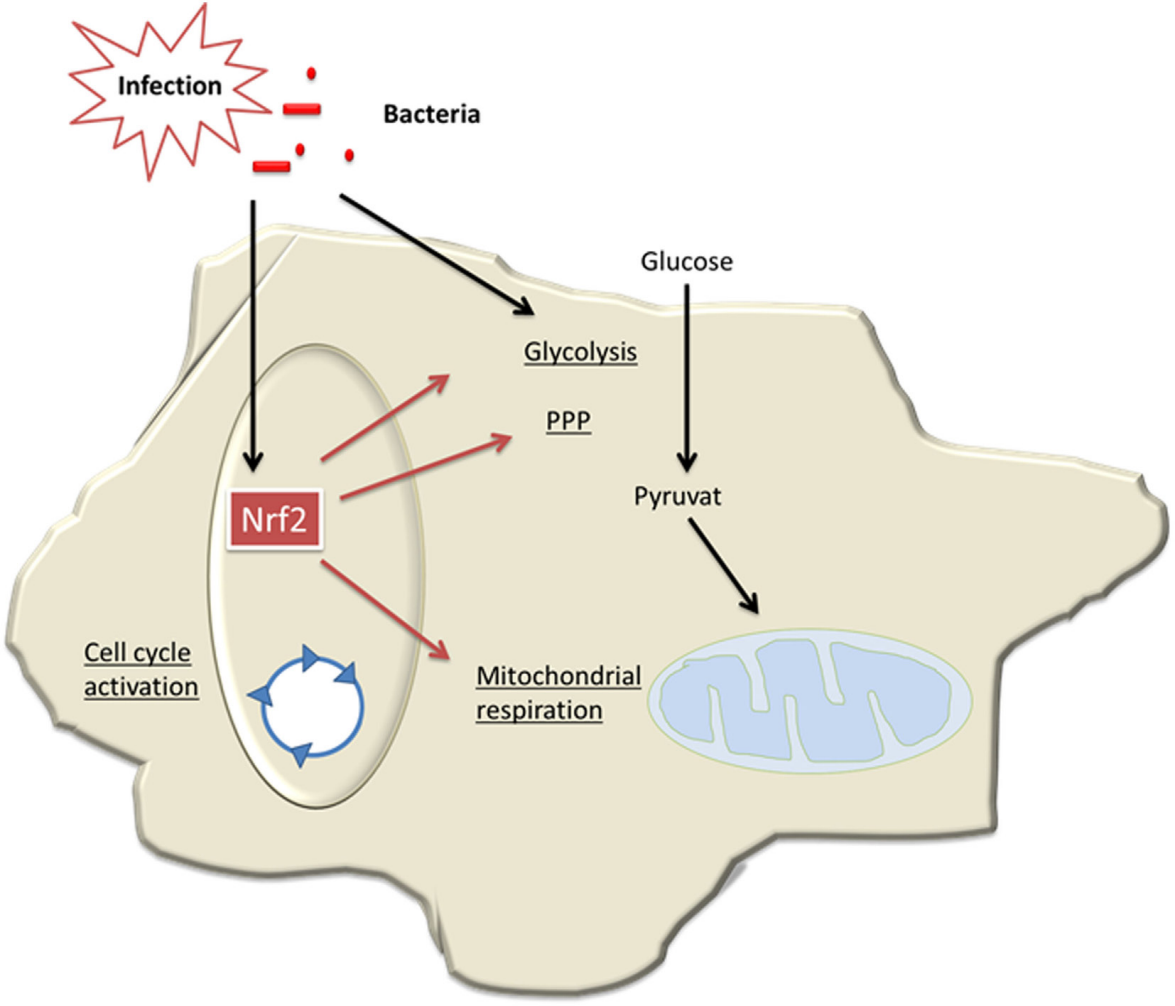
Model of Nrf2-mediated roles in myeloid-derived suppressor cells (MDSCs) in sepsis. During infection and sepsis, Nrf2 is activated by reactive oxygen species (ROS) molecules or TLR pathways. Nrf2 induces cell cycle activation and enhances metabolic activity and thereby contributes to expansion of MDSCs in sepsis.

In conclusion, our data demonstrate for the first time that Nrf2/Keap signaling critically contributes to generation of tolerant MDSCs, which bear an intact cellular energy metabolism and are protective in sepsis (**Figure 8**). Thereby, our study provides new insights into the regulation of MDSCs, a myeloid cell population that might be relevant in trained immunity of the innate immune system (11, 44).

## Ethics Statement

The study was approved by the regional government authorities and animal procedures were performed according to German legislation for animal protection. Permission for the projects was granted by the Regierungspräsident/LANUV Nordrhein-Westfalen.

## Author Contributions

KO developed the study, performed experiments, analyzed data, and wrote the paper. AF generated *VAV^cre^Keap^fl/f^*^l^ mice and performed *in vivo* sepsis experiments. PK, WK, and JB performed experiments. ML and JM performed seahorse assays. IC performed bioinfomatic analyses. BD performed and analyzed microarrays. EV and SB performed experiments. AS, JR, and NW contributed to the writing of the paper. CW and KT developed the study and wrote the paper.

## Conflict of Interest Statement

The authors declare that the research was conducted in the absence of any commercial or financial relationships that could be construed as a potential conflict of interest.

## Abbreviations

ECAR, extracellular acidification rate; DMF, dimethyl fumarate; G6pd, glucose-6-phosphate dehydrogenase; Glut3, glucose transporter 3; Hk, hexokinase; IMCs, immature myeloid cells; Keap1, Kelch ECH associating protein 1; mLN, mesenterial lymph node; MDSC, myeloid-derived suppressor cell; Nrf2, nuclear factor (erythroid-derived 2)-like 2; OCR, oxygen consumption rate; Pgd, phosphogluconate dehydrogenase; Pkm2, pyruvate kinase isozyme M2; PPP, pentose phosphate pathway; ROS, reactive oxygen species; TJL, The Jackson Laboratory Score; Tkt, transketolase; T_reg_ cell, regulatory T cell.
