# Supplementary material for: Nrf2 Is a Central Regulator of Metabolic Reprogramming of Myeloid-Derived Suppressor Cells in Steady State and Sepsis
Source: Front Immunol. 2018 Jul 6;9:1552. doi: 10.3389/fimmu.2018.01552 (PMC6043652; doi:10.3389/fimmu.2018.01552)
Supplement: Supplementary file 1 [file data_sheet_1.PDF]

Supplemental Information

Constitutive Nrf2 activation induces transcriptional reprogramming of myeloid cells and thereby promotes CD11b$^+$Gr-1$^+$ MDSCs accumulation in steady state and sepsis.

Kim Ohl[1,*], Athanassios Fragoulis[2,3,*], Patricia Klemm[1], Julian Baumeister[1], Wiebke Klock[1], Eva Verjans[1,4], Svenja Böll[1,4], Julia Möllmann[5], Michael Lehrke[5], Ivan Costa[6], Bernd Denecke[6], Angela Schippers[1], Johannes Roth[7], Norbert Wagner[1], Christoph Wruck[2,*], Klaus Tenbrock[1,†;*.]

Supplemental Figures

Figure S1

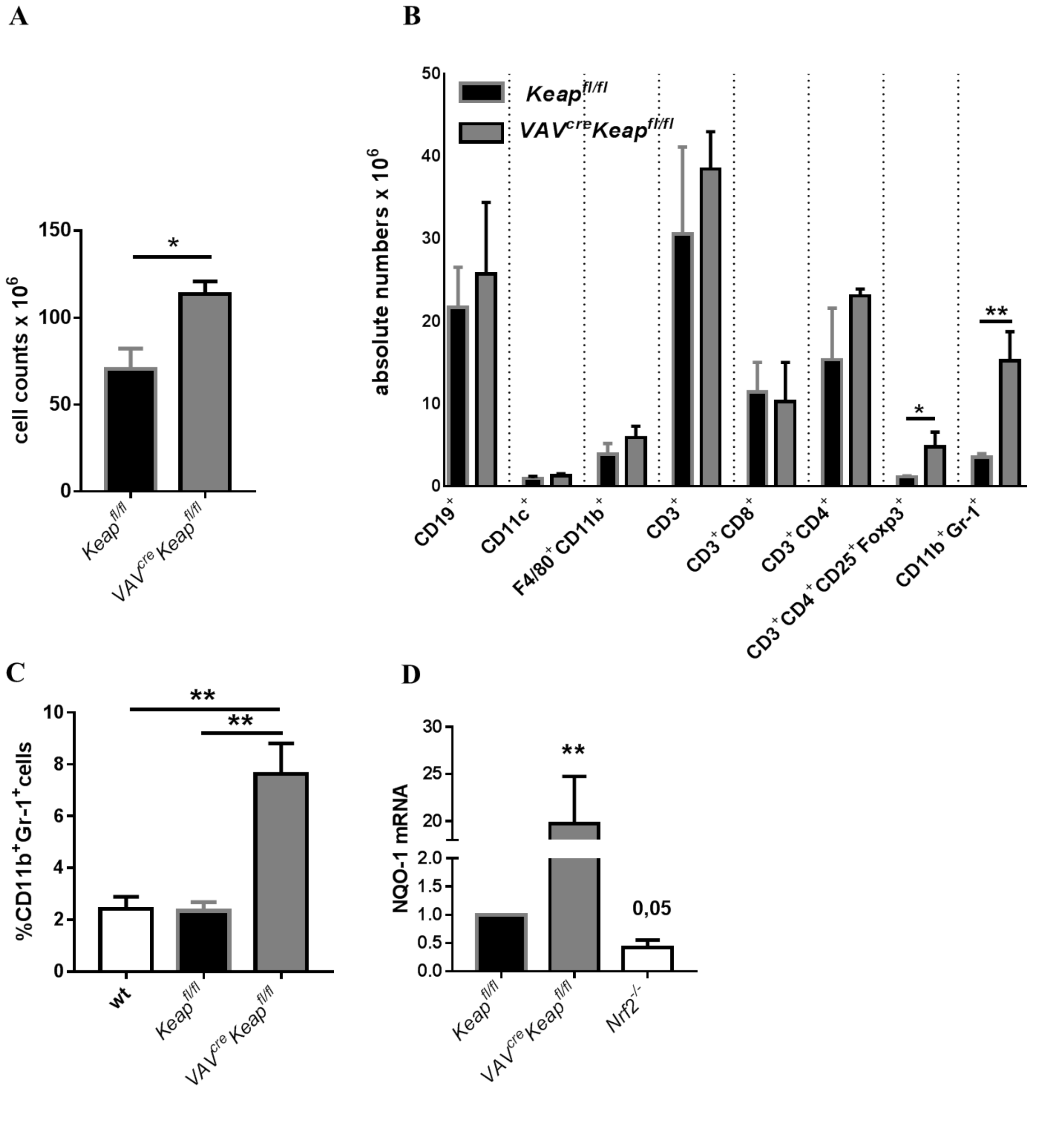

Figure S1: Characterization of mice with a constitutive activation of Nrf2 in immune cells, related to Fig. 1 A) Absolute numbers of cells in spleens from 16 week old WT and *VAV^{cre}Keap$^{fl/fl}$* mice. Bars indicate mean ± SEM of 3 mice per group. B) Absolute numbers of immune cell populations in spleens from 16 week old WT and *VAV^{cre}Keap$^{fl/fl}$* mice. Bars indicate mean ± SEM of 3 mice per group. **C)** Percentages of CD11b$^+$Gr-1$^+$ cells in spleens from 8-12 week old mice. Bars indicate mean and error bars SEM of *Keap$^{fl/fl}$* (n=7), *VAV^{cre}Keap$^{fl/fl}$* (n=7) and WT (n=7) mice. D) Higher expression of NQO-1 in VAV^{cre}Keap$_{fl/fl}$ CD11b$^+$Gr-1$^+$ cells. N-fold expression of NQO-1 in MACS-isolated CD11b$^+$Gr-1$^+$ cells analyzed by RT-qPCR. Bars indicate mean and error bars SEM of *Keap$^{fl/fl}$* (n=7), *VAV^{cre}Keap$^{fl/fl}$* (n=8) and *Nrf2$^{-/-}$* (n=3) mice.

Figure S2

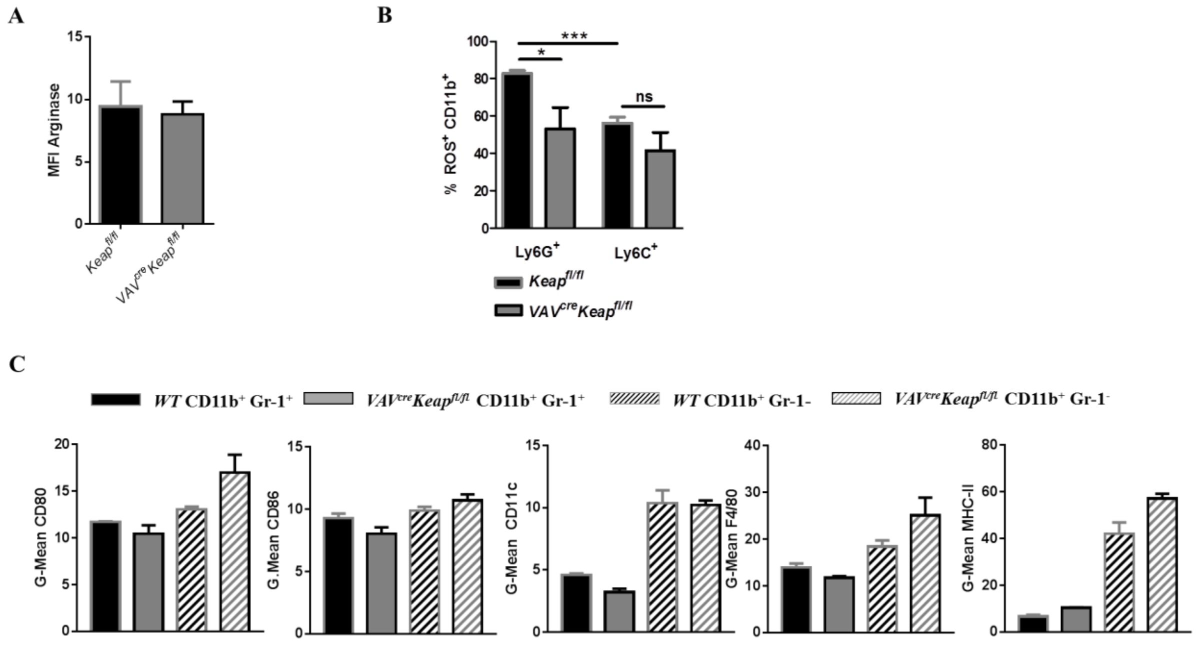

Figure S2: $VAV^{cre}Keap^{fl/fl}$ CD11b^{+}Gr-1^{+} show MDSC characteristics, related to Figure 2.
A) Statistical analysis of arginase-expression in pre-gated CD11b^{+}Gr-1^{+} WT (n =3) and $VAV^{cre}Keap^{fl/fl}$ (n=3) cells. Bars indicate mean of mean fluorescence intensity and error bars SEM. B) Statistical analysis of ROS^{+} cells in pre-gated CD11b^{+}Ly6G^{+} and CD11b^{+}Ly6C^{+} cells from $VAV^{cre}Keap^{fl/fl}$ and $Keap^{fl/fl}$ (n=4) mice, two-tailed unpaired t-test. C) Statistical analysis of expression in pre-gated CD11b^{+}Gr-1^{+} and CD11b^{+}Gr-1^{-} WT (n =3) and $VAV^{cre}Keap^{fl/fl}$ (n=3) mice. Bars indicate mean of mean fluorescence intensity and error bars SEM.

Figure S3

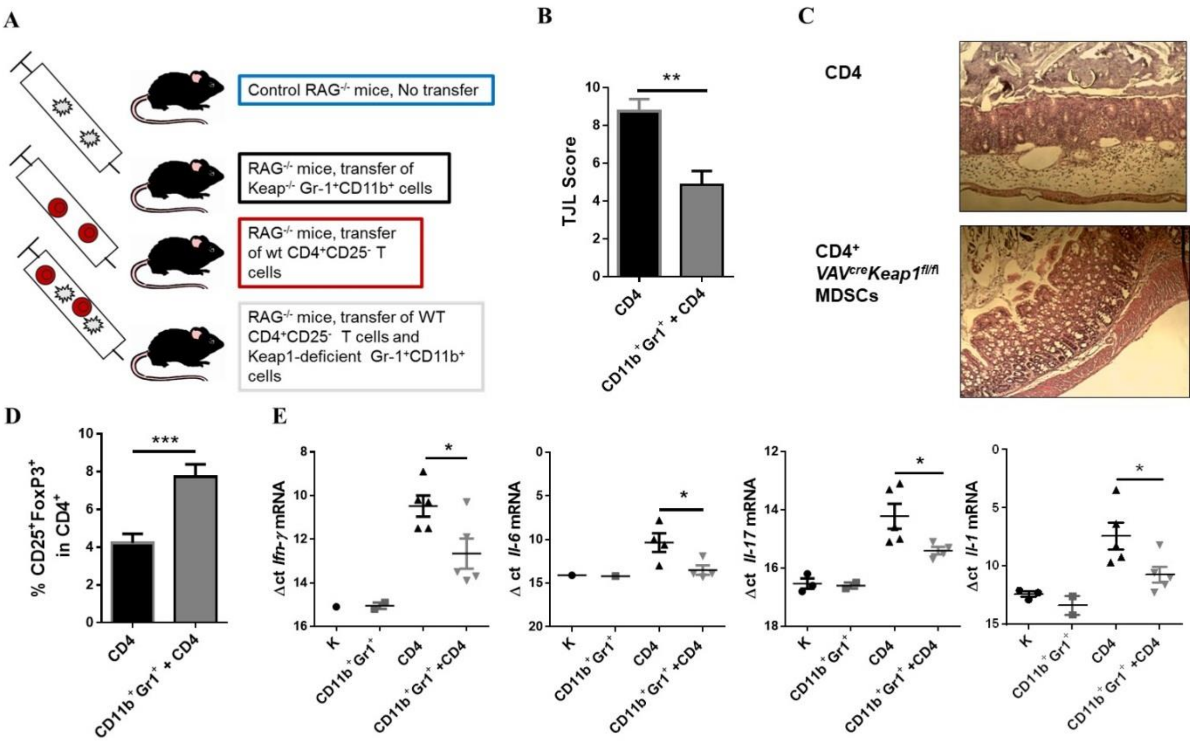

Figure S3: Keap1-deficient MDSCs suppress T-cell mediated colitis, related to Figure 2
A) Model of transfer colitis with MDSC co-transfer. B) Results of histological TJL (The Jackson Laboratory Score) scoring of colon sections. C) Representative photomicrographs of hematoxylin and eosin (H&E)-stained colon sections from $CD4^+$ $CD25^-$ recipients and $CD4^+$ $CD25^-$ and MDSC double recipients. D) Statistical analysis of splenic $CD25^+Foxp3^+$ cells among $CD4^+$ cells assessed by flow cytometry. E) Expression of inflammatory cytokines analyzed by RT-qPCR. Each symbol indicates an individual mouse. Dots represent Δ ct values normalized to β-actin. (Ct levels are inversely proportional to the amount of target nucleic acid in the sample). Error bars represent SEM. For B) and D) bars indicate mean, error bars SEM. Two-tailed unpaired t-tests were used to determine p-values for all statistical analysis.

Figure S4

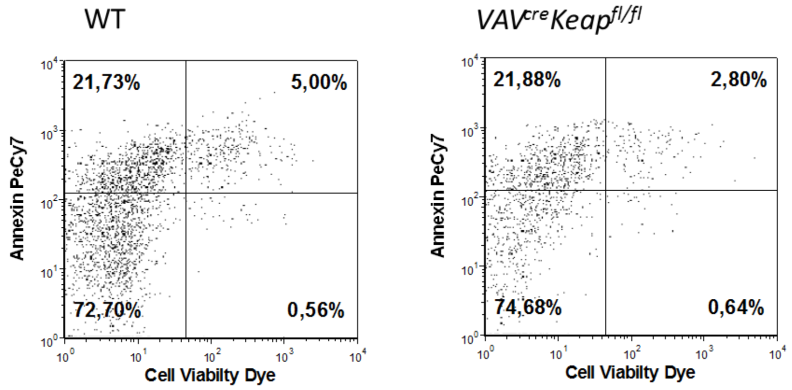

Figure S4: *VAV^{cre}Keap$^{fl/fl}$* CD11b$^+$Gr-1$^+$ cells do not reveal reduced apotosis compared to WT cells, related to Figure 4.

Representative dot plots of the AnnexinV/cell viabilty dye distribution of splenic CD11b$^+$Gr-1$^+$ cells from WT and *VAV^{cre}Keap$^{fl/fl}$* mice.

Figure S5

A

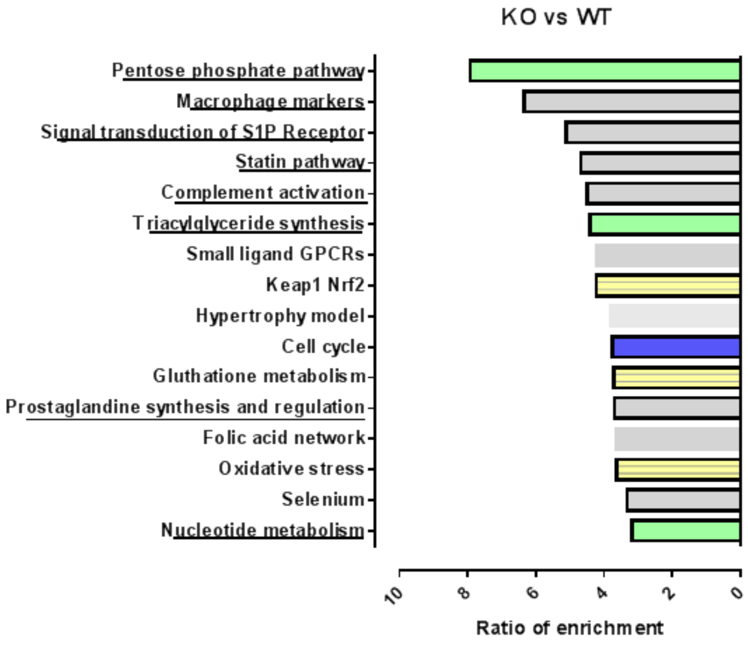

B

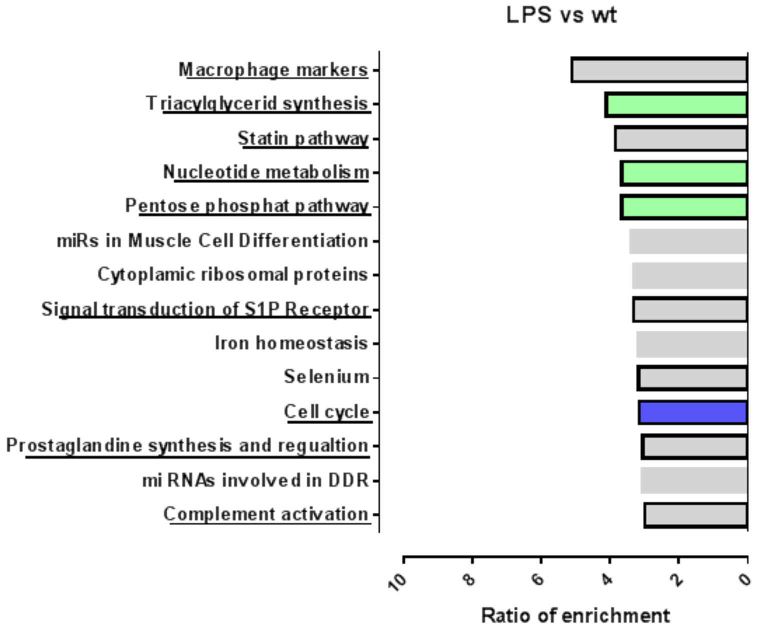

Figure S5: *VAV*^{cre}*Keap*$^{fl/fl}$ and LPS-induced MDSCs both show enrichment of metabolic and cell cycle pathways, related to Figure 6.
Wikipathway analysis showing pathways of differentially expressed genes (ratio < 3, p< 0.05) between *Keap*$^{fl/fl}$ and WT MDSCs (A), as well as between LPS-induced and wildtype MDSCs (B). Gray-framed bars and underlined text mark pathways, which are enriched in both groups. Metabolic pathways (green bars) and cell cycle pathway (blue bar) were enriched in both groups. Known Nrf2 regulated pathways are marked in yellow.

Supplemental Table

Table S1: List of RT-qPCR primers

Name of Genes		Sequence (5'-3')
mGlut3	Forward	CCGCTTCTCATCTCCATTGTCC
	Reverse	CCTGCTCCAATCGTGGCATAGA
mG6PD	Forward	TGCAATTCCGAGATATACCAGGC
	Reverse	ACATGCCAGGCTTCTTGGT
mTkt	Forward	TGTCCCGAAACAAGCCTTCA
	Reverse	ACTCGGTAGCTGGCTTTGTC
mPgd	Forward	GACATTGCACTGATCGGACTG
	Reverse	TCGTTGGCCAAGAAGTCATC
mHk1	Forward	AGGGCGCATTACTCCAGAG
	Reverse	CCCTGTGGGTGTCTTGTGTG
mHk2	Forward	TGATCGCCTGCTTATTCACGG
	Reverse	AACCGCCTAGAAATCTCCAGA
mPkm2	Forward	TCGAGGAACTCCGCCGCCTG
	Reverse	CCACGGCACCCACGGCGGCA
m ß-Actin	Forward	GACTACCTCATGAAGATCCTCACC
	Reverse	TCTCCTTAATGTCACGCACGATT-
Nqo1	Forward	AGAGAGTGCTCGTAGCAGGAT
	Reverse	CTACCCCCAGTGGTGATAGAAA
